# Phylogenetic Analysis and Flower Color Evolution of the Subfamily Linoideae (Linaceae)

**DOI:** 10.3390/plants11121579

**Published:** 2022-06-15

**Authors:** Alejandra Villalvazo-Hernández, Mireya Burgos-Hernández, Dolores González

**Affiliations:** 1 Programa de Posgrado en Botánica, Colegio de Postgraduados, Km 36.Federal Highway Mexico-Texcoco Km 36.5, Montecillo, Texcoco 56230, Mexico; alejandra.vhernandez@alumnos.udg.mx; 2 Red de Biodiversidad y Sistemática, Instituto de Ecología, A.C. Old Road to Coatepec Km 351, El Haya, Xalapa 91073, Mexico; dolores.gonzalez@inecol.mx

**Keywords:** ancestral flower color, evolution, flax lineages, linseed, segregated genera, taxonomy

## Abstract

The taxonomy of the subfamily Linoideae at the intergeneric and section levels has been questioned throughout the years, and the evolution of floral characters remains poorly understood. In particular, the evolution of flower color is still uncertain, despite its ecological importance and being one of the most variable and striking traits in Angiospermae. We evaluated the phylogenetic relationships of the genera and sections and used the phylogeny to reconstruct the ancestral state of flower color. The results suggest reevaluating the taxonomic status of segregated genera and re-incorporating them into *Linum.* Four of the five sections currently accepted were recovered as monophyletic (*Cathartolinum*, *Dasylinum*, *Linum*, and *Syllinum*). We propose accepting the section *Stellerolinon* and reevaluating *Linopsis*, whose representatives were recovered in three separate clades. The ancestral flower color for Linoideae was yellow-white. The flower colors purple and yellow-white were recovered at the deepest nodes of the two main clades. Pink, blue, and red colors were the most recent to evolve. These results appear to be related to diversification events, biogeographical history, and ecological aspects of the subfamily. Our reconstruction constitutes the first plausible scenario that explores the evolution of flower color, leading to new testable hypotheses for future research on the flax group.

## 1. Introduction

Linoideae Arnott is one of the two known subfamilies in Linaceae and the larger of the two. The subfamily is distributed mainly in temperate regions, with the greatest diversity concentrated in the Mediterranean Basin and Southwestern Asia, with some representatives extending to tropical and subtropical latitudes [1,2]. It comprises approximately 210 species in 8 genera [1,3], with *Linum* L. being the most diverse and commercially important [2,4,5]. Recognition of the genus is attributed to the cultivation of *Linum usitatissimum* L., commonly known as flax or linseed [6]. However, some wild species have recently been used for other purposes, such as *Linum perenne* L. and *L. grandiflorum* Desf, which have been used as ornamentals [7,8], and *L. rupestre* (A. Gray) Engelm. ex A. Gray and *L. scabrellum* Planch., which have been used for their medicinal properties [9,10,11].

The species of the genus have great morphological diversity, due in part to the wide range of environments where they live, so species can be difficult to characterize [12,13,14]. Therefore, *Linum* has been subject to several taxonomic changes. For example, although Linoideae was initially organized in the genera *Anisadenia* Wall., *Linum*, *Radiola* Hill, and *Reinwardtia* Dumort, for Planchon [15,16], *Tirpitzia* Hallier f. was segregated from *Reinwardtia* due to the presence of species with tubular corollas characteristic and winged seeds [17]. Furthermore, the monotypic genera *Cliococca* Bab. and *Sclerolinon* C. M. Rogers, as well as the genus *Hesperolinon* (A. Gray) Small with 13 species, originally confined in *Linum*, were segregated from this genus based on morphological characteristics [18,19,20,21].

*Cliococca selaginoides* (Lam.) C. M. Rogers & Mildner was initially described as *Linum selaginoides* Lam. [22], and later, Babington [23], based on the description of cultivated plants at the Cambridge Botanical Garden, considered that there were sufficient characteristics to establish the existence of a new genus: *Cliococca*. Rogers and Mildner [21] reassessed the genera and validated their segregation. Similarly, *Hesperolinon* was also described as a section of *Linum* by Gray [24]. Small [18], based on morphological characters, such as the number of carpels and styles, circumscribed the section at the genus level with *H. californicum* (Benth.) Small. as the type. This status was supported by Sharsmith [19], who detailed the description of this genus. However, its separation from the rest of the flaxes is controversial. Although the genus shows clear morphological differences [18,25], there are no clear differences at the molecular level, and its return to *Linum* has been suggested [1,26]. For its part, *Sclerolinon digynum* (A. Gray) C. M. Rogers was described under the basonym *Linum digynum* (A. Gray). Brewer and Watson [27] and Trelease [28,29] noted the resemblance of this species to *Hesperolinon*. It was the first time that the species was separated from flaxes. A century later, Sharsmith [19] pointed out the need to reassess this species to accurately determine what genus it belongs to and validated *Sclerolinon* as a genus.

The studies of Planchon [15,16], Winkler [30], and Ockendon and Walters [31] constituted the basis for the establishment of the five sections of the *Linum* genus currently recognized in most taxonomic works: *Cathartolinum* (Rchb.) Grised, *Dasylinum* (Planch.) Juz., *Linum* (Planch.) Juz, *Linopsis* (Rchb.), and *Syllinum* Griseb. However, phylogenetic analyses show discrepancies in the validity of some sections. McDill et al. [2], from molecular characters, recovered only two of the five sections, *Dasylinum* and *Cathartolinum*, as natural groups. On the other hand, McDill and Simpson [1], from plastid DNA data, recovered *Linum*, *Dasylinum*, *Syllinum*, and *Cathartolinum* as natural groups. Meanwhile, intending to track the evolution of style polymorphism, Ruiz-Martín et al. [13] and Maguilla et al. [32] recovered only three sections as monophyletic (*Dasylinum*, *Syllinum*, and *Cathartolinum*). Furthermore, other classifications at the section level have recently been evaluated in the group [33]. Therefore, shedding light on this debate is necessary.

The evolution of heterostyly in *Linum* has been extensively studied. Nonetheless, the evolution of other floral features, such as the widely variable color of the corolla among species, has not been studied. Flower color diversity is one of the most striking features of angiosperm radiation. Moreover, it appears to be one of the most evolutive changed traits, often differing between sister species [34,35].

Corolla color spots, patterns, and lines are common across angiosperms and, in ecological terms, are important for mediating plant–animal interactions. For example, color patterns on petals can enhance the pollinator’s ability to detect flowers [36,37], orient it to floral rewards [38], and increase the likelihood of effective pollination [39].

The genetic changes that lead to flower color transitions have been studied in detail [40,41,42]. They are valuable not only for pollination studies [43] but also in evolution. The evolution of character states can be revealed when such states are traced in phylogenetic trees [44]. To clarify the intra- and infrageneric evolutionary relationships of the subfamily Linoideae and contribute for the first time to the knowledge of the evolution of flower color in the group, the aims of this study were: (a) to elucidate the phylogenetic relationships between the genera of Linoideae using molecular characters, (b) to evaluate the phylogenetic position of the five sections of *Linum*, and (c) to reconstruct the ancestral flower color of the lineages of the subfamily.

## 2. Results

### 2.1. Phylogenetic Analysis

**Figure 1:**
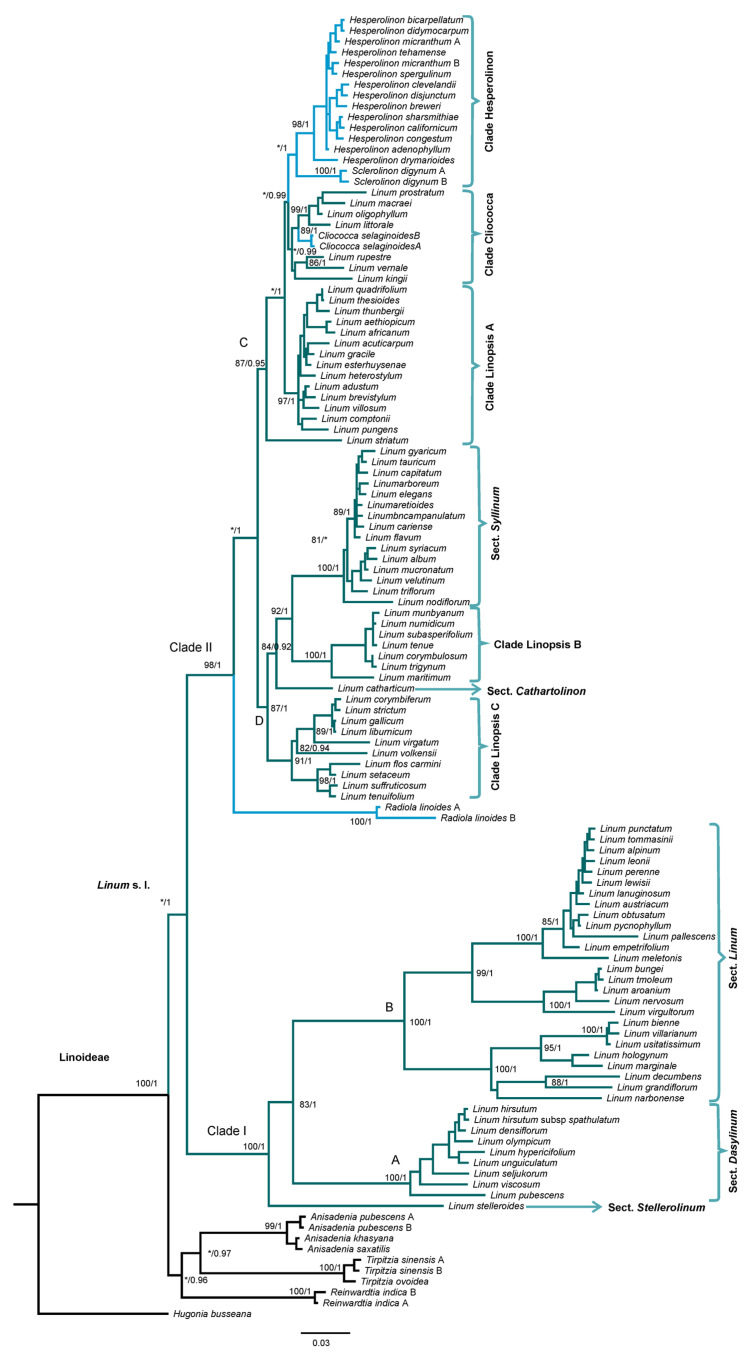
Bayesian Inference tree of Linoideae based on combined plastid (*ndhF*, *matK*, and *trnL-F*) and ITS dataset. The bootstrap values (BS; **left**) and posterior probabilities (PP; **right**) are labeled above the branches. Only support values of BS ≥ 80% and PP ≥ 0.9 are shown. * No support values. A representative of Hugonia (Hugonideae) was used as an outgroup.

The concatenated dataset consisted of 2989 characters, 1631 of which were conserved sites, 1316 were variable sites, and 951 were parsimony-informative. The parsimony analysis (PA) resulted in 240 most-parsimonious trees with a length of 3347, a consistency index (Ci), excluding uninformative characters, of 0.44, and a retention index (Ri) of 0.86. Although significant congruence was observed between topologies derived from Bayesian Inference (BI, Figure 1) and PA analyses (Supplementary Figure S3), with only minor differences in the placement of some taxa, in the topology obtained with parsimony, some poorly resolved clades were recovered. Consequently, hereafter, only the BI tree is described and discussed with the corresponding support values (Figure 1).

The subfamily Linoideae was recovered as a well-supported monophyletic group (BS = 100%, PP = 1), with their members resolved into two major clades. The first one comprised the *Anisadenia* and *Tirpitzia* genera, forming a monophyletic group with moderate support (BS ≤ 80%, PP = 0.97), with *Reinwardtia* as its sister group (BS ≤ 80%, PP = 0.96). The second clade included all Linum species and segregated genera (BS ≤ 80%, PP = 1) resolved in two main clades (I and II).

Clade I (BS = 100%, PP = 1) recovered the section *Dasylinum* as monophyletic (clade A: BS = 100%, PP = 1) and almost all members of the section *Linum*, also as a natural group (clade B: BS = 100%, PP = 1), except for *L. stelleroides* Planch, which was positioned as the basal clade. Within clade B, the position of *L. usitatissimum* showed discrepancies between PA and BI analyses. In the topology obtained with PA, *L. bienne* Mill. was positioned as the sister species of *L. usitatissimum*, although with a low support value (BS < 80%), while in the BI analysis, the cultivated species was positioned as the sister of the clade formed by *L. bienne* and *L. villarianum* Pau., with strong support (PP = 1). Notwithstanding this inconsistency, this clade showed robust support in both analyses (BS = 100%, PP = 1). Additionally, this clade was consistently recovered as a sister of the clade composed of *L. hologynum* Rchb. and *L. marginale* A.Cunn. ex. Planch. (BS = 95%, PP = 1). Interestingly, in the sect. *Linum* (clade B), several species of socioeconomic interest were recovered. For example, in addition to agronomic species, those of ornamental interest, *L. narbonense* L., *L. grandiflorum* Desf., and *L. decumbens* Desf., formed a clade together but with no support (BS < 80%, PP < 0.9). For its part, *L. perenne*, also of ornamental importance, was recovered forming a natural group with *Linum leonii* F. W. Schultz, *L. alpinum* Jacq., *L. tommasinii* (Rchb.), and *L. punctatum* C. Presl., albeit without enough support (BS < 80%, PP < 0.9).

Clade II integrated most of the *Linum* species (31%), as well as the segregated genera. Here, two large well-resolved clades were recovered (C and D), with *Radiola linoides* positioned as their sister group with strong support values (BS = 98%, PP = 1). Clade C (BS = 87%, PP = 0.95) included three minor clades: the clade Hesperolinon (BS < 80%, PP = 1), which integrated the genera *Hesperolinon* and *Sclerolinon*; the clade Cliococca (BS ≤ 80%, PP = 0.99), which was recovered as the sister of clade Hesperolinon and included seven species of the so-called section *Linopsis* [(*Linum oligophyllum* Willd. Ex Schult., *L. littorale* A. St. Hil, *L. macraei* Benth., *L. prostratum* Dombey ex Lam., *L. rupestre* (A. Gray) Engelm. ex A. Gray, *L. vernale* Wooton, and *L. kingii* S. Watson) together with the monospecific genus *Cliococca*; and the clade Linopsis A (BS = 97%, PP = 1), which integrated more representatives of the sect. *Linopsis*.

Clade D (BS = 87%, PP = 1) recovered four minor clades: the section *Syllinum* with strong support values (BS = 100%, PP = 1) and sister to clade Linopsis B, integrating some species from the sect. *Linopsis* (BS = 100%, PP = 1). The clade composed of the monotypic section *Cathartolinum*, which was positioned as basal to the clades Linopsis B and Syllinum (BS = 84%, PP = 0.92), and the fourth clade integrating the remaining species of the sect. *Linopsis* (clade Linopsis C) had high support values (BS = 91%, PP = 1).

### 2.2. Reconstruction of Ancestral Flower Color

**Figure 2:**
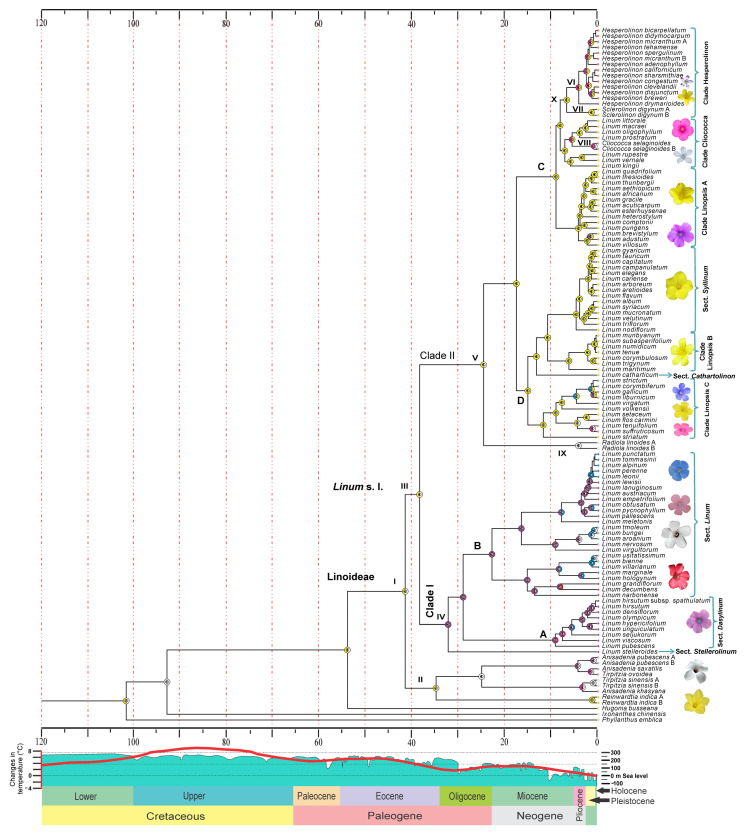
Reconstruction of ancestral states of flower color in the subfamily Linoideae based on S-DIVA analysis. Colored circles at nodes indicate the most likely color state, as estimated by RASP. Roman numerals represent key nodes. The colors at the tips represent the current color of the flower. Global temperature scales and timing of sea-level fluctuations were modified from Zachos et al. [45] and Haq et al. [46], respectively. The geological time scale is in Ma (million years).

The results of the analysis in RASP based on the S-DIVA model (Figure 2) indicated that the flower color of the most recent common ancestor of all living linoids (41.44 Ma: 95% HPD 36.95–47.45 Ma; Supplementary Figure S4) was most likely yellow-white (AC) (node I, Table 1). The same state was recovered for node II (*P* = 0.30), composed of the genera *Anisadenia*, *Tirpitzia*, and *Reinwardtia*, and node III, which corresponds to the species of *Linum* plus segregated genera (=clade *Linum* s.l., *P* = 0.16), which diversified during the Eocene (Priabonian) 34.77 Ma (95% HPD: 20.96–46.61 Ma).

Within the clade *Linum* s.l., node IV (=clade I) corresponds to sects. *Dasylinum* and *Linum*, which recovered purple as the most likely color ancestral state (*P* = 0.32), emerging in the Oligocene at approximately 32.16 Ma (95% HPD 26.33–37.7 Ma). In this same epoch, node V (=clade II, *P* = 0.62, Table 1), which includes sects. *Linopsis*, *Cathartolinum*, and *Syllinum*, as well as segregated genera, showed yellow-white flower color as the most likely ancestral state (Table 1, Figure 2).

Regarding the segregated genera, the clade Hesperolinon (node X) recovered yellow as the ancestral color of the flower during the Miocene (Tortonian, 7.95 Ma), which was also recovered for the genus *Sclerolinon* (node VII, *P* = 1). For the same period, the red color was detected as emerging in the clade of purple flowers in the mid-Miocene (13.5 Ma). The pink color emerged between the end-Miocene and Pleistocene (0.22–5.78 Ma) in the genus *Hesperolinon* (node VI), with the color yellow-pink as the ancestral state (*P* = 0.86), and in Cliococca (node VIII) with the color white-pink (*P* = 1). The above, unlike *Radiola* (node IX), showed white as the most likely state (*P* = 1).

The parsimony ancestral state reconstruction analysis (Supplementary Figure S5) resulted in flower color reconstructions like those obtained with the Bayesian approach (S-DIVA). Minor differences were detected at deep nodes. With parsimony analysis, nodes I, II, III, and V recovered the yellow color as the ancestral state. With RASP, these same nodes retrieved yellow-white as the most likely state. The rest of the nodes similarly recovered the ancestral state in both analyses. According to the parsimony analysis, the pink and white colors of the flowers showed multiple independent origins in subclade II (node V). In subclade I (node IV), the ancestral color was pink-purple.

It is important to note that each of the two large clades into *Linum* s.l. shows a color affinity. In clade I (node IV), corresponding to sections *Dasylinum* and *Linum*, the ancestors show mainly a purple flower color, followed by pink. Clade II (node V) shows a greater distribution of the yellow color among the ancestors and the current species, which is therefore a plesiomorphic state. The pink and blue colors indicate more recent states (~15 and 10 Ma, respectively). For its part, the red color, according to our analysis, is an apomorphic state also of recent origin, since it is present only in two current species, *L. decumbens* and *L. grandiflorum*, both forming a monophyletic group.

## 3. Discussion

### 3.1. Phylogenetic Analysis

Our results support the close relationship between *Anisadenia* and *Tirpitzia*, with *Reinwatdtia* as the sister group, in contrast with McDill and Simpson [1], who recovered *Anisadenia* and *Reinwardtia* as the most closely related genera. Similarly, Ruiz-Martín et al. [13] obtained different results. They recovered *Tirpitzia* and *Reinwardtia* as monophyletic, with *Anisadenia* as the sister genus. The inconsistency in the phylogenetic position of these three genera was pointed out by McDill et al. [2], who concluded that, although the relationship between these genera is uncertain, they are consistently sister to the flax clade.

Within the clade of *Linum* s.l., two large subclades were recovered, consistent with previous phylogenetic work [1,2,13,26,33]. The largest of these subclades integrated *Hesperolinon*, *Sclerolinon*, *Clioccoca*, and *Radiola*. These genera were originally circumscribed within *Linum* [18,19,23] and, later, due to differences in morphological characters, segregated and categorized into the taxonomic rank of the genus. Considering the current circumscription, *Linum* is paraphyletic, which has already been widely highlighted in various phylogenetic studies [1,2,13,26]. However, our results add to those cited above that do not support the current circumscription of the genera, since all of them are nested within the different clades of flax with high support values. Therefore, it has been proposed to reconsider the return of these genera to *Linum*. If so, the genus will be recovered as a natural group.

Thus, *Hesperolinon* is consistently recovered as a monophyletic group nested within one of the large subclades of *Linum*, coinciding with previous works [1,2,13,26,33]. This contrasts significantly with the morphological evidence shown in a recent taxonomic study [25]. In addition, *Hesperolinon* has been highlighted as the only ecological and evolutionarily notable group showing extreme diversification in serpentine soils [1,19,47,48,49]. Geographically, the species of the genus are distributed in a narrow region within the Californian biogeographic province, which has demonstrated not only geographic but also edaphic endemism [4,19,25,26]. Considering these remarkable group differences, the results reported here, and Gray’s [24] proposal, we hypothesize that the *Hesperolinon* species should be treated at the section rank, probably together with *Sclerolinon*, which form a well-supported natural group. This latter genus, originally described as *Linum digynum*, shows important morphological similarities to *Hesperolinon*, which caused it to be transferred to that genus by Sharsmith [19]. However, it was finally elevated to generic rank by Rogers [20] based on the presence of a bicarpellate fruit 4-locular due to the presence of false septa, nuclei with one seed each. Since then, there has been no revision of *Sclerolinon* to give certainty that it is a monotypic genus.

Similarly, we suggest that *Cliococca*, which was initially described as *Linum selaginoides* Lam. until Babington [23] and Rogers and Mildner [21] validated its segregation based on a few morphological characters (presence of decumbent, leafy stems arising from an extensive subterranean pattern, and the presence of indehiscent capsules), should not be considered in the rank of genus. Based on our results, *Cliococca selaginoides* is more closely related to *L. oligophyllum*, *L. littorale*, *L. macraei*, and *L. prostratum*, with high support values. This is also consistent with the different phylogenies of the group [2,13,33]. Returning to the notes made by Rogers and Mildner [21] in their reevaluation of the genus *Clioccoca*, they mentioned that there are sufficient similarities to *Linum*; however, some characters, such as the indehiscent 10-segmented fruit, imbricate corolla, and the unique pollen morphology, suggest that *C. selaginoides* be maintained as a distinct genus from the flaxes. Under these considerations, future work should focus on evaluating the species at the population level and consider the possible hypothesis that it may be a hybrid, even more so when its original description was based on cultivated material from the Cambridge Botanic Garden. In addition, our results also do not support the current *Radiola* circumscription, despite its segregation from *Linum* based on morphological characters [3]. Most likely, it is a section.

Our results support the sectional division of four of the five sections proposed by Ockendon and Walters [31]. These are *Dasylinum*, *Cathartolinum*, and *Syllinum*, and without considering *L. stelleroides*, the sect. *Linum* was also recovered as monophyletic with high support values. Considering our results, we support Yuzepchuk’s [50] proposal to recognize *L. stelleroides* as a monotypic section called *Stellerolinon* Juz. ex Prob. The same has already been considered in recent works [4,33,51]. This is based not only on the fact that the species has a geographical distribution range that is different from the rest (Eastern Asia) but also on the presence of stipulate glands provided with a small stipe, a character that does not appear in any other section. Furthermore, the chromosome number of *L. stelleroides* is 2*n* = 20, different from sects. *Linum* and *Dasylinum*, which have the chromosomal base *n* = 8, 9, or 15 [31,50,52,53]. Meanwhile, all species of the sect. *Linum* lack stipulate glands [2]. For its part, *Linopsis*, the largest of all of the sections, was not recovered as a natural group, and its species were distributed in three clades. According to Planchon [15,16], Winkler [30], Rogers [54], and Ruiz-Martín et al. [13], the characters that describe *Linopsis* are very broad and variable, so a more detailed taxonomic treatment for this section, as well as the inclusion of ecological and biogeographical features, could support the proposal to divide *Linopsis* into independent sections.

Several recent works have shown the importance of identifying and studying the wild relatives of cultivated plants [55,56,57,58]. As sources of new genetic diversity, crop wild relatives have been used for many decades for plant breeding, contributing to a wide range of beneficial agronomic and nutritional traits [55,59]. *Linum ussitatisimum* is not an exception, as it is the species of the greatest importance and was used in ancient times for agronomical purposes in the subfamily, and, in recent years, its production demand has increased [60,61]. Hence, knowing the phylogenetic relationships of this species is essential to explore and estimate the potential use of available resources from its sister species.

Although there is no clarity about the sister group of the cultivated species, our results show a close relationship with *L. villarianun* and *L. bienne*. The relationship of cultivated flax with *L. bienne* was mentioned by McDill et al. [2], McDill and Simpson [1], Schneider et al. [26], Ruiz-Martín et al. [13], Sheidai et al. [62], and, recently, Bolsheva et al. [33], and it has been widely studied since it is considered the old flax wild form cultivated and the wild ancestor of the modern cultivated flax [2,12,63,64]. Something important is that, except for the work by Ruiz-Martín et al. [13], no studies have included *L. villarianum* in their analyses. The phylogenetic closeness of this last species with *L. ussitatisimum* represents an opportunity to focus efforts on studying it under the premise that it represents an important potential resource. However, this analysis only includes 54% of the species of the subfamily and 73% of the sect. *Linum*. Therefore, it is likely that by including the remaining species, the phylogenetic relationships of *L. usitatissimum* will change. As shown in this analysis, just by including one more species, the relationships with cultivated flax are uncertain and weak. It is urgent and essential to represent the remaining 46% of Linoideae. Including the rest of the taxa will surely shed light on this controversy, and the hypotheses proposed here will be tested.

Although they attract less attention, the rest of the species that have gained interest for their uses should not be left out. Among those, *L. grandiflorum* and *L. narbonense* stand out, reported as ornamentals [8], and our results recovered them forming a clade together with *L. decumbens*. These three species are the sister group of the clade that integrates cultivated flax. *Linum perenne* and *L. lewisii*, also reported as ornamentals [65,66,67], were recovered with a close relationship and as sister species to *L. punctatum*, *L. tommasinii*, *L. alpinum*, and *L. leonii*. All of them belong to the sect. *Linum*. The present work is the first to incorporate *L. narbonense* into a phylogenetic analysis, whose position had not been evaluated before and which has also been reported for medicinal use [68]. Other species of the genus have been recognized for their traditional uses. *Linum rupestre*, for example, is used in some localities in the state of Chiapas, Mexico, as a medicinal plant [11]. This species was recovered in the present analysis in a monophyletic group with *L. vernale* and *L. kingii*. Despite the importance of these and other flax species in medicine, phytochemistry, and ornamentals, several of them have not been a focus of interest, and little or nothing has been explored, not only at the molecular level but also in terms of their potential for use.

### 3.2. Reconstruction of Ancestral Flower Color

The diversity of the colors of flowers is one of the most striking characteristics of the radiation of angiosperms since this character has allowed us to identify and measure the interaction with animals [34,35,36,37]. The subfamily Linoideae has been characterized by the inclusion of a great diversity of colors in the corolla, which has translated into the ornamental interest in several of its species [5,6,14,25,54,69]. Our study provides the first tentative evidence that the color of the ancestral flowers of Linoideae was most likely yellow-white. Flower color transitions in Linoideae have not received much attention from an evolutionary perspective either. McDill et al. [2] were the first to formally recognize a clade of blue flowers and another clade of yellow flowers in *Linum* s.l., data that were perpetuated in the subsequent publications of the group. However, this identification was only based on the flower colors of the current species, without delving further into its evolutionary significance.

Based on our analyses of the reconstruction of ancestral states, yellow-white was the plesiomorphic state (41.44 Ma: 95% HPD 36.95–47.45 Ma), from which the purple flower evolved in the Oligocene at 32.16 Ma (clade IV: sects. *Linum*, *Dasyllinum*, and *Stellerolinon*), followed by blue and red colors in the late Miocene (~13 Ma), with pink as the most recent color to evolve (end-Miocene–Pleistocene), coinciding with the period of the greatest radiation in the subfamily. Previous studies have implicated flower color shifts in speciation [70,71]. To the extent that flower color plays a role in speciation events, it is important to determine what evolutionary forces underlie its divergence.

The emergence of the purple color (node IV) in one of the major clades in *Linum* s.l. in the late Eocene and Oligocene coincided with a period of low temperatures, which, in the Northern Hemisphere, became too cold [48,72]. According to the ancestral areas reconstructed by Maguilla et al. [32], in this period, the most recent common ancestor to *Linum* s.l. inhabited the Western Palearctic (i.e., Europe, North Africa, northern and central Arabian Peninsula, and part of temperate Asia). This cold event in the north probably caused the expansion of the lineages, since this climatic change promoted the establishment of communities dominated by temperate vegetation, mainly herbaceous species [73]. In addition, this could have favored the establishment of flax species, since they have shown a preference for habitats dominated by grasses and small herbaceous plants [1,2,13].

The Miocene was characterized by greater aridity that allowed the further expansion of sclerophyll shrublands and woodlands [48,74,75]. In this geological time, the red color evolved from a purple ancestor between the mid-Miocene (~13.5 Ma) and late Miocene (~7 Ma). In that same epoch, the blue color also emerged from different ancestors throughout clade I. At this point, the most likely ancestral area of the lineages was the Western Palearctic, and currently, this clade is essentially Eurasian [32]. In the middle of the Miocene, the starting phase of global cooling and rapid aridification led to the expansion of grassland and xeric vegetation [75,76]. The impact of the dry climate had important effects not only on the Western Palearctic but also across the entire Northern Hemisphere [77]. These events led to important diversification events, supported by growing evidence for species in the western Mediterranean [78,79,80]. These changes were likely what caused the greatest radiation of flax species in the Mediterranean Basin, producing its current status as a Linoideae hotspot.

The effects were also significant in Africa, where the summer monsoon was drastically reduced by the narrowing of the Tethys Sea during the Tortonian age (11.6–7.2 Ma) [81]. These changes altered the composition and distribution of Northern Hemisphere flora [76,82] and coincided with the emergence of the two important lineages in clade C (American and African lineages). This cladogenetic event was congruent with several geological events. The rise of the African lineage (clade Linopsis A) [13,32] was concordant with the connection formed between Africa and south-western Asia due to the collision of the Afro-Arabian plate with the Iranian and Anatolian plates [83]. Furthermore, the main collision with Eurasia resulted in the closure of the Tethys Sea with the formation of the Gomphoterium land bridge during the mid-Miocene, causing African and Eurasian biota to interact [84]. On the other hand, the colonization of the American continent (American lineage: North American = clade Hesperolinon + South American = clade Clioccoca) was congruent with an existing connection between North America and Eurasia. It had been assumed that lineages with divergence times between Eurasia and America younger than the Eocene would not have passed across a North Atlantic land bridge (NALB) but rather across the Bering Strait [85]. However, a review of Neogene sedimentary rocks with plants from Iceland [86,87,88,89,90] revealed rich warm-temperate to temperate flora that lasted at least until 9–8 Ma. This was supported by various studies on temperate flora that corroborated a migration between America and Eurasia via the NALB during the Miocene and up to the Pliocene [86,88,90,91,92,93]. At the end of the Miocene and the middle Pliocene (7.95–3.75 Ma), the pink color arose in the North America and South America lineages independently, both evolving from ancestors with yellow flowers. It is important to highlight that the yellow and pink colors dominate the flowers (Figure 2) of the American lineages, and yellow dominate the African lineages [32]. Results can be influenced by the time of separation of both continents with their respective biogeographical events. These conditions probably favored the diversification and fixation of the pink color through the current species.

The pink color also emerged in the Eurasian species *L. viscosum* and *L. pubescens* in clade A in the late Miocene–Pliocene and more recently in the Asian genera *Anisadenia* and *Tirpitzia* during the Pliocene–Pleistocene (~2.5 Ma). This last epoch was characterized by glacial and interglacial cycles that resulted in the evolution and migration of many plant lineages, favoring their diversification to alternate environments [79]. It is currently known that the Mediterranean Basin served as a refuge for many species during the Tertiary and Quaternary glaciations and as a source for the subsequent colonization of adjacent areas as Asia regions [94]. However, although the geological and paleoclimatic events mentioned could suggest a close relationship between the biogeographical history of the group and the color of the flowers, they do not explain why the pleisiomorphic yellow state is mainly maintained throughout clade II and the purple state is maintained in clade I. Here, it is likely that pollinators played a more important role.

Flower color transitions usually accompany a shift in pollination mode [95]. Observations on pollination in *Linum* or sister genera are scarce, scattered in the literature, and/or have not been updated [96,97,98,99]. The little that is known about the group has been generalized from those studies, and it is mentioned that *Linum* flowers are typically pollinated by insects such as honeybees, bumblebees, flies, and butterflies [13,99]. It is known that, due to differences in preferences, different functional groups of pollinators may select different flower colors [95,100,101]. However, many floral radiations exhibit a remarkable variety of colors despite members sharing the same functional group of pollinators [102,103,104,105]. The above suggests that the pollinator shift model does not fully account for the diversity of colors across angiosperms [106]. In addition, it is important to mention that we examined the color evolution of the corolla based on human perception and not the ultraviolet light (UV) spectrum that pollinators can detect, mainly insects [107]. It has been suggested that the evolution of human-visible patterns is associated with the evolution of larger flowers, but the evolution of UV patterns is correlated with the evolution of smaller flowers [108]. Therefore, we suggest complementing this work from an ecological perspective, since little is known about the types of pollinators and details about pollination in Linoideae. Moreover, color vision can vary among insect species, so this information cannot be generalized [109].

A few studies have examined the tempo of discrete changes in flower color, such as gains or losses of pigmentation [110,111]. According to our results, the most likely scenario is that pigmentation was gained in a speciation event from an ancestral lineage of white-yellow flowers. According to Ng and Smith [112], the appearance of a gain of pigmentation mutant in an ancestral population could also lead to the emergence of a new lineage if this trait allows or even promotes dispersal to a new region. This is consistent with the geological period with the greatest diversification of Linoideae, which coincides with dispersal events to new geographical areas of several lineages, and provides an initial assessment of the possible role of flower color in dispersal to new habitats. Nonetheless, we cannot rule out a pollinator-mediated scenario where a sub-population disperses to a new region with a different pollinator fauna that selects for colored flowers [111,113]. Studies about pollinators are needed to test this hypothesis. Once this knowledge is acquired, it will be possible to know and understand how Linoideae’s pollinators, especially insects, perceive colors and thus understand plant-pollinator interactions more precisely in this group.

Our study provides a novel picture of the flower color of the most recent ancestor of all living Linoideae and the earliest steps of color polymorphisms. The ancestral flower color for Linoideae was yellow-white, with the purple color dominating clade I ancestors and the yellow color dominant throughout clade II, suggesting a scenario closely related to the biogeographical history of the group and its pollinators. However, new progress in reconstructing the evolutionary steps and integrating breakthroughs in evo-devo and ecological research is still necessary. Likewise, the taxonomic status of the segregated genera was explored, and as a result, we propose here that they be reconsidered so that they are returned to *Linum* and that the current sectional status is reevaluated. These results are a contribution toward an understanding of floral color pattern diversity and evolution, as well as the systematics of the subfamily Linoideae.

## 4. Material and Methods

### 4.1. Taxon Sampling

The taxonomic diversity of Linoideae was represented by 451 accessions of 113 species covering the eight genera of the subfamily (Supplementary Table S1). *Hugonia busseana* Engl. (subfamily Hugonioideae) was included as an outgroup along with *Phyllanthus emblica* L. and *Ixonanthes chinensis* (Hook. & Arn.) Champ., representing the two sister families of Linaceae: Phyllanthaceae and Ixonanthaceae [114]. Sequences from these taxa were retrieved from GenBank (Supplementary Table S1) and correspond to four DNA regions: the nuclear ribosomal DNA ITS1-5.8S-ITS2 (ITS) region, Maturase K (*matK*), NADH-dehydrogenase subunit F (*ndhF*), and the intergenic spacer between tRNA^Leu^ and tRNA^Phe^ (*trnL-trnF*). Sequences were aligned using PhyDe software [115] with the Muscle algorithm [116], followed by a final adjustment by visual inspection.

### 4.2. Phylogenetic Analysis

Phylogenetic analyses were performed separately for ITS and cpDNA (*matK*, *ndhF*, and *trnL-trnF*) and in combination (ET = ITS + cpDNA). Analyses were performed with *H. busseana* (subfamily Hugonioideae) as the outgroup. The congruence of the phylogenetic signals from ITS and cpDNA was evaluated by visual comparison of their respective topologies. Furthermore, an incongruence length difference (ILD) test [117], implemented in PAUP v4.0a168 [118] as the partition-homogeneity test between the ITS and cpDNA datasets, was conducted. The partition homogeneity test revealed that partitions were homogeneous (*P* > 0.05). There were no strongly conflictive topologies found among molecular data partitions (Supplementary Figures S1 and S2). Therefore, we concatenated both datasets for further analyses and discussion.

Phylogenetic reconstruction was carried out using Parsimony Analysis (PA) and Bayesian Inference (IB) approaches. Of the regions recovered from GenBank, 5.98% were missing in some taxa; hence, they were coded as “missing data (?)”. PA was performed with the heuristic search implemented in TNT v1.5 [119] with 100 iterations with the TBR (Tree Bisection Reconnection) algorithm, retaining 100 trees per iteration. Gaps were recorded as missing. The shortest trees obtained were saved for calculating the strict consensus tree. Statistical branch support was determined by bootstrap (BS) analysis running 1000 sampling replicates with replacement and collapsing those clades with a value lower than 50% through the “Cutoff” option.

The IB analyses were carried out in MrBayes v3.2.7a [120] with the Markov Chain Monte Carlo (MCMC) technique. Model parameters were fixed according to the values obtained with jModeltest v2.1.10 [121] for each of the matrices and selected with Akaike’s criterion (AIC) [122]. The models used were TVM + G for cpDNA and GTR + I + G for the concatenated matrix and ITS dataset. Each MCMC analysis was run for 10 million generations with four MCMC chains—one cold and three heated—starting from different random points in the parameter space with a discarded burn-in of 25% and sampled every 1000th generation. The outgroup was never forced to be monophyletic during searches. Nodes with posterior probabilities (PP) > 60% were retained in the majority-rule consensus tree. Finally, the trees obtained were visualized and edited in FigTree v1.4 [123].

### 4.3. Reconstruction of Ancestral Flower Color

Reconstruction of ancestral states using model-based methods requires a phylogenetic tree with branch lengths proportional to time, i.e., a timeline. The precedent is to avoid bias by assuming a strict correlation between molecular and morphological evolutionary rates. Therefore, molecular dating analyses were conducted using BEAST v1.10.1 [124] with the individual datasets (Supplementary Material Table S2) and concatenated matrix under an uncorrelated lognormal relaxed-clock model. Based on the results of the study by Xi et al. [125], two secondary calibration points were selected. The first of them was used to calibrate the root node of Phyllanthaceae + [(Ixonanthaceae + Linaceae) under a normal distribution (mean = 102.5; SD = 4.03). The second was used to calibrate the stem node of Linaceae + Ixonanthaceae (mean = 90; SD = 8.65). A third calibration point from fossil pollen grain data unequivocally attributed to *Linum* from the late Eocene from the Ebro basin in northeastern Spain [126,127] was used to calibrate the minimum stem node divergence of this genus under a lognormal distribution (mean = 1; SD = 1; offset = 35.55).

The model parameter implemented for the molecular clock implemented was GTR + I + G for all datasets. This was the closest model to those calculated according to the AIC in jModelTest. Tree priors were modeled with a birth–death process, which models speciation and extinction patterns. Three independent MCMC analyses were run for ITS and ET, each with 50,000,000 generations. For the cpDNA, 120,000,000 generations were carried out in four independent analyses. In all cases, sampling was performed every 1000th generation. The convergence and stationarity of the estimated parameter values were assessed according to effective sample size (ESS > 200), traces, and Bayesian density plots using Tracer v1.7. (Germany) [128]. The log files were combined using LogCombiner. A maximum-clade-credibility (MCC) tree representing the maximum a posteriori topology, with mean divergence times and a posterior probability limit of 0.9, was calculated after the removal of 10% of trees as burn-in using TreeAnnotator v1.10.4. (New Zeland) [129]. Finally, the trees were visualized with FigTree v1.4. (UK) [123].

We recorded the flower colors of 112 species of Linoideae using herbarium data, systematic and taxonomic studies, regional floras, and a database [5,6,13,54,130,131,132,133]. We did not use any general family descriptions or make any assumptions that all species of a genus share the same character state. The Bayesian method of ancestral state reconstruction (GTR + I + G model) Statistical Dispersal-Vicariance Analysis (S-DIVA) [134], implemented in RASP v3.2.1 (China) (Reconstruct Ancestral State in Phylogenies) [135], was performed to reconstruct the ancestral flower color. Each terminal in the tree was coded for six color states divided into the following categories: yellow, blue, white, purple, red, and pink. Flower colors with the highest probability value are indicated by the colored circle at each node of the tree, and the probability values are given in Table 1. For comparison, the ancestral color of flowers was also reconstructed using parsimony, as implemented in Mesquite v2.75 (Canada) [136]. All characters were treated as unordered.

## Figures and Tables

**Table 1:** Divergence time estimates from BEAST analysis and probabilities of the ancestral color of the flower estimated with the Statistical Dispersal-Vicariance Analysis (S-DIVA) in RASP for key nodes of Linoideae in analysis of concatenated data. 95% HPD = 95% highest posterior density; Ma = million years; AC = area code; *P* = probability value.

Code	Node	Mean (Ma)	95% HPD (Ma)	AC	*P*	Color Code
I	Linoideae	41.44	36.95–47.45	AC	0.25	A = Yellow C = White D = Purple AC = Yellow + White AD = Yellow + Purple AF = Yellow + Pink CD = White + Purple CF = White + Pink
				CD	0.15	
II	*Anisadenia* + *Reinwardtia* + *Tirpitzia*	34.77	20.96–46.61	AC	0.30	
				C	0.20	
III	*Linum* s.l.	38.32	35.64–42.91	AC	0.16	
				C	0.15	
IV	Subclade I (sections *Dasylinum* + *Linum* + *Stellerolinum*)	32.16	26.33–37.7	D	0.32	
				CD	0.18	
V	Subclade II (sections *Linopsis + Syllinum + Cathartolinum +* segregated genera)	24.49	16.29–33.68	AC	0.62	
				A	0.15	
VI	*Hesperolinon*	3.75	1.95–5.78	AF	0.86	
				AD	0.14	
VII	*Sclerolinon*	1.19	0.25–2.36	A	1.00	
VIII	*Cliococca*	0.22	0–0.61	CF	1.00	
IX	*Radiola*	4.01	1.15–8.11	C	1.00	
X	Clade Hesperolinon (*Hesperolinon + Sclerolinon*)	6.55	4.14–9.36	A	0.84	
				AF	0.15	

## Data Availability

Not applicable.

## Supplementary Materials

The following supporting information can be downloaded at: https://www.mdpi.com/article/10.3390/plants11121579/s1. Table S1: GenBank accessions numbers for *ndhF*, *matK*, *trnL*-*F*, and ITS; Table S2: Divergence time estimates from BEAST analysis for key nodes of the Linoideae subfamily based on partitioned data; Figure S1: Phylogenetic relationships of the Linoideae subfamily based on combined plastid DNA: (A) Parsimony tree. (B) Bayesian Inference tree; Figure S2: Phylogenetic relationships of the Linoideae subfamily based on ITS: (A) Parsimony tree. (B) Bayesian Inference tree; Figure S3: Parsimony analysis tree of Linoideae based on combined plastid and ITS dataset; Figure S4: Beast maximum clade credibility tree of Linoideae inferred from combined ITS, *ndhF*, *matK*, and *trnL*-*F*; Figure S5: Reconstruction of ancestral states of flower color in the subfamily Linoideae based on Most Parsimonious Reconstruction.

## References

[1] McDill J., Simpson B.B. (2011). Molecular phylogenetics of Linaceae with complete generic sampling and data from two plastid genes. Bot. J. Linn. Soc..

[2] McDill J., Repplinger M., Simpson B.B., Kadereit W.J. (2009). The phylogeny of *Linum* and Linaceae subfamily Linoideae, with implications for their systematics, biogeography, and evolution of heterostyly. Syst. Bot..

[3] Dressler S.M., Repplinger M., Bayer C., Kubitzki K. (2014). Linaceae. Flowering Plants. Eudicots.

[4] Melnikova N.V., Kudryavtseva A.V., Zelenin A.V., Lakunina V.A., Yurkevich O.Y., Speranskaya A.S., Snezhkina A.V. (2014). Retrotransposon-based molecular markers for analysis of genetic diversity within the genus. Linum. BioMed Res. Int..

[5] Rzedowski J., Calderón de Rzedowski G. (1992). Linaceae. Flora del Bajío y de Regiones Adyacentes.

[6] Rzedowski J., Calderón de Rzedowski G. (1994). Fascículo 5. Linaceae. Flora del Valle de Tehuacán-Cuicatlán.

[7] Touré A., Xu X.M. (2010). Flaxseed lignans: Source, biosynthesis, metabolism, antioxidant activity, bio-active components, and health benefits. Compr. Rev. Food Sci. Food Saf..

[8] Singh K.K., Mridula D., Rehal J., Barnwal P. (2011). Flaxseed: A potential source of food, feed and fiber. Crit. Rev. Food Sci. Nutr..

[9] Lautié E., Quintero R., Fliniaux M.A., Villarreal M.L. (2008). Selection methodology with scoring system: Application to Mexican plants producing podophyllotoxin related lignans. J. Ethnopharmacol..

[10] Alonso-Castro J.A., Villarreal M.L., Sálazar-Olivo A.L., Gomez-Sanchez M., Dominguez F., Garcia-Carranca A. (2011). Mexican medicinal plants used for cancer treatment: Pharmacological phytochemical and ethnobotanical studies. J. Ethnopharmacol..

[11] Barrera-Robles P.J., Burgos-Hernández M., Ruíz-Acevedo A.D., Castillo-Campos G. (2020). La familia Linaceae en México: Estado actual y perspectivas. Bot. Sci..

[12] Fu Y.-B., Peterson G., Diederichsen A., Richards K.W. (2002). RAPD analysis of genetic relationships of seven flax species in the genus *Linum* L.. Genet. Resour. Crop Evol..

[13] Ruiz-Martín J., Santos-Gally R., Escudero M., Midgley J.J., Pérez-Barrales R., Arroyo J. (2018). Style polymorphism in *Linum* (Linaceae): A case of Mediterranean parallel evolution?. J. Plant Biol..

[14] Burgos-Hernández M., Castillo-Campos G. (2019). Contribución al conocimiento del género *Linum* (Linaceae) en Veracruz, México. Acta Bot. Mex..

[15] Planchon J.E. (1847). Sur la famille des Linées. Lond. J. Bot..

[16] Planchon J.E. (1848). Sur la famille des Linées. Lond. J. Bot..

[17] Hallier H. (1921). Beitrëge zur Kenntnis der Linaceae (DC. 1819) Dumort. Beih. Bot. Cent. Abt. 1.

[18] Small J.K. (1907). Linaceae. N. Am. Flora.

[19] Sharsmith H.K. (1961). The genus *Hesperolinon* (Linaceae). Univ. Calif. Publ. Bot..

[20] Rogers C.M. (1966). *Sclerolinon*, a new genus in the Linaceae. Madroño.

[21] Rogers C.M., Mildner R. (1971). The reevaluation of the genus *Cliococca* (Linaceae) of South America. Rhodora.

[22] Lamarck J.B. (1791). Encyclopédie Méthodique, Botanique 1.

[23] Babington C.C. (1842). Description of a new genus of Lineae. Trans. Linn. Soc. Lond..

[24] Gray A. (1865). *Linum* sect. Hesperolinon. Proc. Am. Acad. Arts.

[25] González-Velasco J., Burgos-Hernández M., Galván-Escobedo I.G., Castillo-Campos G. (2022). Taxonomic update of the flax family in Mexico. Phytotaxa.

[26] Schneider A.C., Freyman W.A., Guilliams C.M., Springer Y.P., Baldwin B.G. (2016). Pleistocene radiation of the serpentine—Adapted genus *Hesperolinon* and other divergence times in Linaceae (Malpighiales). Am. J. Bot..

[27] Brewer W.H., Watson S., Gray A. (1876). Botany [of California].

[28] Trelease W. (1887). A revision of North American Linaceae. Trans. Acad. Sci. St. Louis.

[29] Trelease W. (1897). Linaceae. Synoptical Flora of North America.

[30] Winkler H. (1931). Linaceae. Die Natürlichen Pflanzenfamilien.

[31] Ockendon D.J., Walters S.M. (1968). Linum. Flora Europea.

[32] Maguilla E., Escudero M., Ruiz-Martín J., Arroyo J. (2021). Origin and diversification of flax and their relationship with heterostyly across the range. J. Biogeogr..

[33] Bolsheva N.L., Melnikova N.V., Dvorianinova E.M., Mironova L.N., Yurkevich O.Y., Amosova A.V., Krasnov G.S., Dimitriev A.A., Muravenko O.V. (2022). Clarification of the position of *Linum stelleroides* Planch. within the phylogeny of the genus *Linum* L.. Plants..

[34] Wesselingh R.A., Arnold M.L. (2000). Pollinator behaviour and the evolution of Louisiana iris hybrid zones. J. Evol. Biol..

[35] Martén-Rodríguez S., Fenster C.B., Agnarsson I., Skog L.E., Zimmer E.A. (2010). Evolutionary breakdown of pollination specialization in a Caribbean plant radiation. New Phytol..

[36] Lehrer M., Horridge G.A., Zhang S.W., Gadagkar R. (1995). Shape vision in bees: Innate preference for flower-like patterns. Philos. Trans. R. Soc. Lond. B Biol. Sci..

[37] Koski M.H., Ashman T.L. (2014). Dissecting pollinator responses to a ubiquitous ultraviolet floral pattern in the wild. Funct. Ecol..

[38] Manning A. (1956). The Effect of Honey-Guides. Behaviour.

[39] Hansen D.M., van der Niet T., Johnson S.D. (2012). Floral signposts: Testing the significance of visual ‘nectar guides’ for pollinator behaviour and plant fitness. Proc. R. Soc. B.

[40] Streisfeld M.A., Rausher M.D. (2009). Altered trans-regulatory control of gene expression in multiple anthocyanin genes contributes to adaptive flower color evolution in *Mimulus aurantiacus*. Mol. Biol. Evol..

[41] Smith S.D., Rausher M.D. (2011). Gene loss and parallel evolution contribute to specie difference in flower color. Mol. Biol. Evol..

[42] Zhang B., Liu C., Yao X., Wang F., Wu J., King G.J., Liu F. (2015). Disruption of a carotenoid cleavage dioxygenase 4 gene converts flower colour from white to yellow in *Brassica* species. New Phytol..

[43] Eaton D.A.R., Fenster C.B., Hereford J., Huang S., Ree R.H. (2012). Floral diversity a community structure in *Pedicularis* (Orobanchaceae). Ecology.

[44] Lawson D.A., Rands S.A. (2018). The evolution of floral guides: Using a genetic algorithm to investigate the evolution of floral cue arrangements. Biol. J. Linn. Soc. Lond..

[45] Zachos J., Pagani M., Sloan L., Thomas E., Billups K. (2001). Trends, rhythms, and aberrations in global climate 65 Ma to present. Science.

[46] Haq B.U., Hardenbol J.A.N., Vail P.R. (1987). Chronology of fluctuating sea levels since the Triassic. Science.

[47] Raven P.H., Axelrod D.I. (1978). Origin and relationships of the California flora. Univ. Calif. Publ. Bot..

[48] Anacker B.L., Whittall J.B., Goldberg E.E., Harrison S.P. (2011). Origins and consequences of serpentine endemism in the California flora. Evolution.

[49] Springer Y.P. (2009). Do extreme environments provide a refuge from pathogens? A phylogenetic test using serpentine flax. Am. J. Bot..

[50] Yuzepchuk S.V., Shishkin B.K., Bobrov E.G. (1949). Genus *Linum*-Linaceae Dumort. Flora SSSR (Flora of the Soviet Union).

[51] Bolsheva N.L., Melnikova N.V., Kirov I.V., Speranskaya A.S., Krinitsina A.A., Dmitriev A.A., Muravenko O.V. (2017). Evolution of blue-flowered species of genus *Linum* based on high-throughput sequencing of ribosomal RNA genes. BMC Evol. Biol..

[52] Martzenitzina K.K. (1927). The chromosomes of some species of the genus *Linum* L.. Bull. Appl. Bot. Genet. Plant Breed..

[53] Petrova A.V. (1927). IOPB Chromosome number reports XXXV. Taxon.

[54] Rogers C.M. (1982). The systematics of *Linum* sect. Linopsis. Plant Syst. Evol..

[55] Castañeda-Alvarez N.P., Khoury C.K., Achicanoy H.A., Bernau V., Dempewolf H., Eastwood R.J., Toll J. (2016). Global conservation priorities for crop wild relatives. Nat. Plants.

[56] Von Wettberg E.J., Chang P.L., Başdemir F., Carrasquila-Garcia N., Korbu L.B., Moenga S.M., Bedada G., Greenlon A., Moriuchi K.S., Singh V. (2018). Ecology and genomics of an important crop wild relative as a prelude to agricultural innovation. Nat. Commun..

[57] Nair K.P. (2019). Utilizing crop wild relatives to combat global warming. Adv. Agron..

[58] Vincent H., Hole D., Maxted N. (2022). Congruence between global crop wild relative hotspots and biodiversity hotspots. Biol. Conserv..

[59] Brozynska M., Furtado A., Henry R.J. (2016). Genomics of crop wild relatives: Expanding the gene pool for crop improvement. Plant Biotechnol. J..

[60] Eyres L. (2015). Flaxseed fibre—A functional superfood?. Food N. Z..

[61] Kaur P., Waghmare R., Kumar V., Rasane P., Kaur S., Gat Y. (2018). Recent advances in utilization of flaxseed as potential source for value addition. OCL.

[62] Sheidai M., Darini S., Talebi S.M., Koohdar F., Ghasemzadeh-Baraki S. (2019). Molecular systematic study in the genus *Linum* (Linaceae) in Iran. Acta Bot. Hung..

[63] Tammes T. (1928). The genetics of the genus *Linum*. Bibliogr Genet..

[64] Allaby R.G., Peterson G.W., Merriwether A., Fu Y.B. (2005). Evidence of the domestication history of flax (*Linum usitatissimum*) from genetic diversity of the sad2 locus. Theor. Appl. Genet..

[65] Maxted N., Scholten M., Codd R., Ford-Lloyd B. (2007). Creation and use of a national inventory of crop wild relatives. Biol. Conserv..

[66] Cullis J.O. (2011). Diagnosis and management of anaemia of chronic disease: Current status. Br. J. Haematol..

[67] Tork D.G., Anderson N.O., Wyse D.L., Betts K.J. (2019). Domestication of perennial flax using an ideotype approach for oilseed, cut flower, and garden performance. Agronomy.

[68] Mohammed M.M., Christensen L.P., Ibrahim N.A., Awad N.E., Zeid I.F., Pedersen E.B., Jensen K.B., Colla P.L. (2009). Anti-HIV-1 activities of the extracts from the medicinal plant *Linum grandiflorum* Desf. In Proceedings of 4th Conference on Research and Development of Pharmaceutical Industries (Current Challenges). Med. Aromat. Plant Sci. Biotechnol..

[69] Rogers C.M. (1975). Relationships of *Hesperolinon* and *Linum* (Linaceae). Madroño.

[70] Bradshaw H.D., Wilbert S.M., Otto K.G., Schemske D.W. (1995). Genetic mapping of floral traits associated with reproductive isolation in monkey flowers (*Mimulus*). Nature.

[71] Van der Niet T., Johnson S.D. (2012). Phylogenetic evidence for pollinator driven diversification of angiosperms. Trends Ecol. Evol..

[72] Miller K.G., Fairbanks R.G., Mountain G.S. (1987). Tertiary oxygen isotope synthesis, sea level history and continental margin erosion. Paleoceanogr. Paleoclimatol..

[73] Tiffney B.H., Manchester S.R. (2001). The use of geological and paleontological evidence in evaluating plant phylogeographic hypotheses in the Northern Hemisphere Tertiary. Int. J. Plant Sci..

[74] Bush M., Flenley J., Gosling W. (2011). Tropical Rainforest Responses to Climatic Change.

[75] Herbert T.D., Lawrence K.T., Tzanova A., Peterson L.C., Caballero-Gill R., Kelly C.S. (2016). Late Miocene global cooling and the rise of modern ecosystems. Nat. Geosci..

[76] Milne R.I., Abbott R.J. (2002). The origin and evolution of tertiary relict floras. Adv. Bot. Res..

[77] Weijermars R. (1988). Neogene tectonics in the Western Mediterranean may have caused the Messinian Salinity Crisis and an associated glacial event. Tectonophysics.

[78] Casimiro-Soriguer R., Talavera M., Balao F., Terrab A., Herrera J., Talavera S. (2010). Phylogeny and genetic structure of *Erophaca* (Leguminosae), a East-West Mediterranean disjunct genus from the Tertiary. Mol. Phylogenet. Evol..

[79] Fernández-Mazuecos M., Jiménez-Mejías P., Rotllan-Puig X., Vargas P. (2014). Perspectives in plant ecology, evolution and systematics narrow endemics to Mediterranean islands: Moderate genetic diversity but narrow climatic niche of the ancient, critically endangered *Naufraga* (Apiaceae). Perspect. Plant Ecol. Evol. Syst..

[80] García-Castaño J.L., Terrab A., Ortiz M.A., Stuessy T.F., Talavera S. (2014). Patterns of phylogeography and vicariance of *Chamaerops humilis* L. (Palmae). Turk. J. Bot..

[81] Zhang Z., Ramstein G., Schuster M., Li C., Contoux C., Yan Q. (2014). Aridification of the Sahara Desert caused by Tethys Sea shrinkage during the Late Miocene. Nature.

[82] Medail F., Diadema K. (2009). Glacial refugia influence plant diversity patterns in the Mediterranean Basin. J. Biogeogr..

[83] Popov S., Rögl F., Rozanov A., Steininger F.F., Shcherba I., Kovac M. (2004). Lithological-paleogeographic maps of Paratethys. Late Eocene to Pliocene. Cour. Forschungsinst. Senckenberg.

[84] Rögl F. (1999). Mediterranean and Paratethys. Facts and hypotheses of an Oligocene to Miocene paleogeography (short overview). Geol. Carpath..

[85] Donoghue M.J., Smith S.A. (2004). Patterns in the assembly of temperate forests around the Northern Hemisphere. Philos. Trans. R. Soc. B Biol. Sci..

[86] Grímsson F., Denk T. (2005). *Fagus* from the Miocene of Iceland: Systematics and biogeographical considerations. Rev. Palaeobot. Palynol..

[87] Grímsson F., Denk T. (2007). Floristic turnover in Iceland from 15 to 6 Ma extracting biogeographical signals from fossil floral assemblages. J. Biogeogr..

[88] Grímsson F., Denk T., Símonarson L.A. (2007). Middle Miocene floras of Iceland—The early colonization of an island?. Rev. Palaeobot. Palynol..

[89] Grímsson F., Denk T., Zetter R. (2008). Pollen, fruits, and leaves of *Tetracentron* (Trochodendraceae) from the Cainozoic of Iceland and western North America and their palaeobiogeographic implications. Grana.

[90] Denk T., Grimsson F., Zetter R., Símonarson L. (2011). Late Cainozoic Floras of Iceland: 15 Million Years of Vegetation and Climate History in the Northern North Atlantic.

[91] Akhmetiev M.A., Bratzeva G.M., Giterman R.E., Golubeva L.V., Moiseyeva A.I. (1978). Late Cainozoic Stratigraphy and Flora of Iceland.

[92] Denk T., Grímsson F., KvaČek Z. (2005). The Miocene floras of Iceland and their significance for late Cainozoic North Atlantic biogeography. Bot. J. Linn. Soc..

[93] Milne R.I. (2004). Phylogeny and biogeography of *Rhododendron* subsection *Pontica*, a group with a tertiary relict distribution. Mol. Phylogenet. Evol..

[94] Thompson J.D. (2020). Plant Evolution in the Mediterranean: Insights for Conservation.

[95] Fenster C.B., Armbruster W.S., Wilson P., Dudash M.R., Thomson J.D. (2004). Pollination syndromes and floral specialization. Annu. Rev. Ecol. Evol. Syst..

[96] Williams I.H., Martin A.P., Clark S.J. (1990). Pollination requirements of linseed (*Linum usitatissimum*). J. Agric. Sci..

[97] Kearnsaf1 C.A., Inouye D.W. (1994). Fly pollination of *Linum lewish* (Linaceae). Am. J. Bot..

[98] Gürbüz B. (1999). Determination of cross-pollination in flax (*Linum usitatissimum*) using different experimental designs. J. Agric. Sci..

[99] Lebel M., Obolski U., Hadany L., Sapir Y. (2018). Pollinator-mediated selection on floral size and tube color in *Linum pubescens*: Can differential behavior and preference in different times of the day maintain dimorphism?. Ecol. Evol..

[100] Whittall J.B., Hodges S.A. (2007). Pollinator shifts drive increasingly long nectar spurs in columbine flowers. Nature.

[101] Campbell D.R., Bischoff M., Lord J.M., Robertson A.W. (2010). Flower color influences insect visitation in alpine New Zealand. Ecology.

[102] Armbruster W.S. (2002). Can indirect selection and genetic context contribute to trait diversification? A transition-probability study of blossom-colour evolution in two genera. J. Evol. Biol..

[103] Cooley A.M., Carvallo G., Willis J.H. (2008). Is floral diversification associated with pollinator divergence? Flower shape, flower colour and pollinator preference in Chilean *Mimulus*. Ann. Bot..

[104] Smith C.I., Godsoe W.K., Tank S., Yoder J.B., Pellmyr O. (2008). Distinguishing coevolution from covicariance in an obligate pollination mutualism: Asynchronous divergence in Joshua tree and its pollinators. Evolution.

[105] Paget-Seekins J. (2012). *Ribes* (Grossulariaceae) Pollination in Northern California: Strong Overlap in Visitor Assemblages Despite Floral Diversity. Master’s Thesis.

[106] Muchhala N., Johnsen S., Smith S.D. (2014). Competition for hummingbird pollination shapes flower color variation in Andean Solanaceae. Evolution.

[107] Song B.M., Lee C.H. (2018). Toward a mechanistic understanding of color vision in insects. Front. Neural Circuits.

[108] Koski M.H. (2020). Macroevolution of flower color patterning: Biased transition rates and correlated evolution with flower size. Front. Plant Sci..

[109] Van der Kooi C.J., Stavenga D.G., Arikawa K., Belušič G., Kelber A. (2021). Evolution of insect color vision: From spectral sensitivity to visual ecology. Annu. Rev. Entomol..

[110] Smith S.D., Miller R.E., Otto S.P., FitzJohn R.G., Rausher M.D. (2010). The effects of flower color transitions on diversification rates in morning glories (Ipomoea subg. Quamoclit, Convolvulaceae). Darwin’s Heritage Today, Proceedings of the Darwin 200 Beijing International Conference, Beijing, China, 24–26 October 2009.

[111] Sobel J.M., Streisfeld M.A. (2013). Flower color as a model system for studies of plant evo-devo. Front. Plant Sci..

[112] Ng J., Smith S.D. (2014). How traits shape trees: New approaches for detecting character state-dependent lineage diversification. J. Evol. Biol..

[113] Waser N.M., Campbell D.R. (2004). Ecological speciation in flowering plants. Adaptive Speciation.

[114] Chase M.W., Christenhusz M.J.M., Fay M.F., Byng J.W., Judd W.S., Soltis D.E., Mabberley D.J., Sennikov A.N., Soltis P.S., The Angiosperm Phylogeny Group (2016). An update of the Angiosperm Phylogeny Group classification for the orders and families of flowering plants: APG IV. Bot. J. Linn. Soc..

[115] Müller J., Müller K., Neinhuis C., Quandt D. (2005). PhyDE-Phylogenetic Data Editor. http://www.phyde.de.

[116] Edgar R.C. (2004). MUSCLE: Multiple sequence aligment with high accurancy and high troughput. Nucleic Acids Res..

[117] Farris J.S., Kallersjo M., Kluge A.G., Bult C. (1995). Constructing a significance test for incongruence. Syst. Biol..

[118] Swofford D.L. PAUP* Phylogenetic Analysis Using Parsimony Version 4.0a168. http://paup.sc.fsu.edu.

[119] Goloboff P.A., Farris J.S., Nixon K.C. (2008). TNT, a free program for phylogenetic analysis. Cladistics.

[120] Ronquist F., Teslenko M., van der Mark P., Ayres D.L., Darling A., Höhna A.S., Larget B., Liu L., Suchard M.A., Huelsenbeck J.P. (2012). MRBAYES 3.2: Efficient Bayesian phylogenetic inference and model selection across a large model space. Syst. Biol..

[121] Darriba D., Taboada G.L., Doallo R., Posada D. (2012). jModelTest 2: More models, new heuristics and parallel computing. Nat. Methods.

[122] Akaike H. (1974). A new look at the statistical model identification. IEEE Trans. Automat. Contr..

[123] Rambaut A. FigTree v1.4. http://tree.bio.ed.ac.uk/software/figtree/.

[124] Suchard M.A., Lemey P., Baele G., Ayres D.L., Drummond A.J., Rambaut A. (2018). Bayesian phylogenetic and phylodynamic data integration using BEAST 1.10. Virus Evol..

[125] Xi Z., Ruhfel B.R., Schaefer H., Amorim A.M., Sugumaran M., Wurdack K.J., Endress P.K., Matthews M.L., Stevens P.F., Mathews S. (2012). Phylogenomics and a posteriori data partitioning resolve the *Cretaceous angiosperm* radiation Malpighiales. Proc. Natl. Acad. Sci. USA.

[126] Punt W., Den Breejen P. (1981). Linaceae. Rev. Palaeobot. Palynol..

[127] Cavagnetto C., Anadón P. (1996). Preliminary palynological data on floristic and climatic changes during the Middle Eocene-Early Oligocene of the eastern Ebro Basin, northeast Spain. Rev. Palaeobot. Palynol..

[128] Rambaut A., Drummond A.J., Xie D., Baele G., Suchard M.A. (2018). Posterior summarization in Bayesian phylogenetics using Tracer 1.7. Virus Evol..

[129] Rambaut A., Drummond A. (2016). TreeAnnotator v.2.4.3. http://beast.community/treeannotator.

[130] Rogers C.M. (1963). Yellow flowered species of *Linum* in Eastern North America. Brittonia.

[131] Rogers C.M. (1964). Yellow-flowered *Linum* (Linaceae) in Texas. Sida.

[132] Rogers C.M. (1968). Yellow-flowered species of *Linum* in Central America and western North America. Brittonia.

[133] POWO, Plants of the World Online Facilitated by the Royal Botanic Gardens, Kew. http://www.plantsoftheworldonline.org/.

[134] Yan Y., Harris A.J., He X. (2010). S-DIVA (Statistical Dispersal-Vicariance Analysis): A tool for inferring biogeographic histories. Mol. Phylogenet. Evol..

[135] Yu Y., Blair C., He X.J. (2020). RASP 4: Ancestral State Reconstruction Tool for Multiple Genes and Characters. Mol. Biol. Evol..

[136] Maddison W.P., Maddison D.R. (2011). Mesquite: A Modular System for Evolutionary Analysis. Version 2.75. http://mesquiteproject.org.

