# Supplementary material for: Phylogenetic Analysis and Flower Color Evolution of the Subfamily Linoideae (Linaceae)
Source: Plants (Basel). 2022 Jun 15;11(12):1579. doi: 10.3390/plants11121579 (PMC9231132; doi:10.3390/plants11121579)
Supplement: Supplementary file 1 [file plants-11-01579-s001.zip › Supplementary material.pdf]

plants

MDPI

Supplementary material

Table S1. GenBank accessions numbers for *ndhF*, *matK*, *trnL-F* and ITS.

Genera	Species		*ndhF*	*matK*	*trnL-trnF*	ITS
**Hugonoideae**						
Hugonia	*busseana*		FJ160773	HM544087	FJ160857	MK066674
**Linoideae**						
Anisadenia	*pubescens* A		FJ160772	HM544078	FJ160856	FJ169513
Anisadenia	*pubescens* B		MK090350		MK066784	MK066671
Anisadenia	*khasyana*			HM544077		
Anisadenia	*saxatilis*			HM544079		
Cliococca	*selaginoides* A		MK090351	HM544080	MK066785	KT453457
Cliococca	*selaginoides* B		FJ160774		FJ160858	MK066672
Hesperolinon	*adenophyllum*			FJ011121	FJ011277	KT453359
Hesperolinon	*bicarpellatum*			FJ011124	FJ011280	
Hesperolinon	*breweri*			FJ011131	FJ011287	
Hesperolinon	*californicum*			FJ011145	FJ011301	KT453387
Hesperolinon	*clevelandii*			HM544083	FJ011308	KT453392
Hesperolinon	*congestum*			FJ011158	FJ011314	KT453398
Hesperolinon	*didymocarpum*			FJ011163	FJ011319	KT453403
Hesperolinon	*disjunctum*			HM544084	KT453495	KT453413
Hesperolinon	*drymarioides*			HM544085	FJ011327	KT453419
Hesperolinon	*micranthum* A *		FJ160775	HM544086	KT453507	KT453440
Hesperolinon	*micranthum* B *		MK090352	HM544086	MK066786	MK066673
Hesperolinon	*sharsmithiae*			FJ011184	FJ011339	KT453446
Hesperolinon	*sperguLinum*			FJ011188	FJ011344	KT453451
Hesperolinon	*tehamense*			FJ011193	FJ011349	KT453456
Radiola	*linoides* A		FJ160815	HM544118	FJ160899	MK066777
Radiola	*linoides* B		MK090456	HM850983	MK066889	FJ169534
Reinwardtia	*indica* A		MK090457	AB048380	MK066890	MK066778
Reinwardtia	*indica* B		KX526972	HM544119	FJ160898	FJ169514
Sclerolinon	*digynum* A		FJ160787	HM544122	FJ160871	FJ169541
Sclerolinon	*digynum* B		MK090458	AB233792	MK06689	MK066779
Tirpitzia	*ovoidea*			HM544123		
Tirpitzia	*sinensis* A		MK090459	HM544124	MK066892	MK066780
Tirpitzia	*sinensis* B		FJ160816	FJ160855	FJ160900	FJ169515
		**Sección**				
Linum	*acuticarpum*	*Linopsis*	MK090354	MK098985	MK066788	MK066675
Linum	*adustum*	*Linopsis*	MK090355	MK098986	MK066789	MK066676
Linum	*aethiopicum*	*Linopsis*	MK090356	MK098987	MK066790	MK066677

Linum	*africanum*	*Linopsis*	FJ160777	MK098988	FJ160861	MK066678
Linum	*album*	*Syllinum*	FJ160792	FJ160831	FJ160876	MK066679
Linum	*alpinum*	*Linum*	MK090359	MK098989	MK066793	MK066680
Linum	*arboreum*	*Sylllinum*	FJ160793	HM544100	FJ160877	MK066681
Linum	*aretioides*	*Syllinum*	MK090361	MK098991	MK066795	MK066682
Linum	*aroanium*	*Linum*	MK098992	MK090362	MK066796	MK066683
Linum	*austriacum*	*Linum*	FJ160799	FJ160838	FJ160883	KY661907
Linum	*bienne*	*Linum*	FJ160797	HM544102	KT453509	KT453458
Linum	*brevistylum*	*Linopsis*	MK090368	MK098997	MK066801	MK066689
Linum	*bungei*	*Linum*	MK090369	MK098998	MK066802	MK066690
Linum	*campanulatum*	*Syllinum*	MK090370	MK098999	MK066803	MK066691
Linum	*capitatum*	*Syllinum*	MK090337	MK099000	MK066804	MK066692
Linum	*cariense*	*Syllinum*	MK090372	MK099001	MK066805	MK066693
Linum	*catharticum*	*Cathartolinum*	FJ160796	HM544103	FJ160880	MK066694
Linum	*comptonii*	*Linopsis*	FJ160778	HM544104	FJ160862	MK066695
Linum	*corymbiferum*	*Linopsis*	MK090375	MK099004	MK066808	MK066696
Linum	*corymbulosum*	*Linopsis*	MK090376	MK099005	MK066809	MK066697
Linum	*decumbens*	*Linum*	MK090377	MK099007	MK066811	MK066699
Linum	*densiflorum*	*Dasylinum*	MK090380	MK099009	MK066813	MK066701
Linum	*elegans*	*Syllinum*	MK090381	MK099010	MK066814	MK066702
Linum	*empetrifolium*	*Linum*	MK099012	MK090382	MK066915	MK066815
Linum	*esterhuysenae*	*Linopsis*	FJ160779	MK099013	MK066817	MK066705
Linum	*flavum*	*Syllinum*	FJ160794	HM544105	FJ160878	MK066706
Linum	*flos-carmini*	*Linopsis*	MK090426	MK099043	MK066819	MK066747
Linum	*gallicum*	*Linopsis*	MK090386	MK099015	MK066819	MK066707
Linum	*gracile*	*Linopsis*	FJ160776	MK099016	FJ160860	MK066708
Linum	*grandiflorum*	*Linum*	FJ160798	KX783704	FJ160882	MK066709
Linum	*gyaricum*	*Syllinum*	MK090389	MK099017	MK066822	MK066710
Linum	*heterostylum*	*Linopsis*	MK090390	MK099018	MK066823	MK066711
Linum	*hirsutum*	*Dasylinum*	FJ160788	HM544106	FJ160872	MK066712
Linum	*hirsutum* subsp. *spathulatum*	*Dasylinum*	MK099044	MK090427	MK066860	MK066748
Linum	*hologynum*	*Linum*	MK090392	MK099020	MK066825	MK077713
Linum	*hypericifolium*	*Dasylinum*	FJ160789	HM544107	FJ160873	MK066714
Linum	*kingii*	*Linopsis*	FJ160780	FJ160823	FJ160864	MK066715
Linum	*lanuginosum*	*Linum*	MK090395	MK099022	MK066828	MK066716
Linum	*leonii*	*Linum*	MK090396	MK099023	MK066829	MK066717
Linum	*lewisii**	*Linum*	FJ160800	FJ160839	KT453510	KT453459
Linum	*liburnicum*	*Linopsis*	MK090398	MK099024	MK066831	MK066719
Linum	*littorale*	*Linopsis*	FJ160781		FJ160865	MK066720
Linum	*macraei*	*Linopsis*	MK090400		MK066833	MK066721

Linum	*marginale*	*Linum*	FJ160804	FJ160843	FJ160888	MK066723
Linum	*maritimum*	*Linopsis*	FJ160811	FJ160850	MK066835	MK066723
Linum	*meletonis*	*Linum*	MK090403	MK099025	MK066836	MK066724
Linum	*mucronatum*	*Sylinum*	KJ194521	MK099027	MK066838	MK066726
Linum	*mumbyanum*	*Linopsis*	MK090406	MK099028	MK066839	MK066727
Linum	*narbonense*	*Linum*	MK090407	HM544109	MK066840	MK066728
Linum	*nervosum*	*Linum*	MK090408	HM544110	MK066841	MK066729
Linum	*numidicum*	*Linopsis*	MK090410	MK099031	MK066843	MK066731
Linum	*nodiflorum*	*Linopsis*	MK090410	FJ160834	MK066843	FJ169539
Linum	*obtusatum*	*Linum*	MK090411	MK099032	MK066844	MK066732
Linum	*oligophyllum*	*Linopsis*	FJ160783	HM544111	FJ160867	MK066733
Linum	*olympicum*	*Dasylinum*	MK099034	MK090413	MK066846	MK066734
Linum	*pallescens*	*Linum*	FJ160801	FJ160840	FJ160885	MK066735
Linum	*perenne*	*Linum*	FJ160802	HQ593346	FJ160886	MK066736
Linum	*prostratum*	*Linopsis*		MK090416	MK066849	MK066737
Linum	*pubescens*	*Dasylinum*	FJ160790	FJ160829	FJ160874	MK066738
Linum	*pungens*	*Linopsis*	MK090420	MK099037	MK066853	MK066741
Linum	*punctatum*	*Linum*	MK090418	MK099035	MK066852	MK066740
Linum	*pycnophyllum*	*Linum*	MK090421	MK099038	MK066854	MK066742
Linum	*quadrifolium*	*Linopsis*	MK090422	MK099039	MK066855	MK066743
Linum	*rupestre**	*Linopsis*	FJ160785	HM544113	FJ160869	KT453460
Linum	*seljukorum*	*Dasylinum*	MK090424	MK099041	MK066857	MK066745
Linum	*setaceum*	*Linopsis*	MK090425	MK099042	MK066858	MK066746
Linum	*stelleroides*	*Linum*	FJ160805	MH660026	FJ160889	MK066749
Linum	*striatum*	*Linopsis*	FJ160786	FJ160825	MK066863	MK066750
Linum	*strictum*	*Linopsis*	FJ160806	MK099045	FJ160890	MK066751
Linum	*subasperifolium*	*Linopsis*	MK090431	MK099046	MK066864	MK066752
Linum	*suffruticosum*	*Linopsis*	FJ160807	MK099048	MK066866	MK066753
Linum	*syriacum*	*Syllinum*	MK090434	MK099049	MK066867	MK066755
Linum	*tauricum*	*Syllinum*	MK090435	MK099050	MK066868	MK066756
Linum	*tenue*	*Linopsis*	FJ160808	MK099053	FJ160892	MK066759
Linum	*tenuifolium*	*Linopsis*	FJ160809	MK966947	FJ160893	MK066761
Linum	*thesioides*	*Linopsis*	MK090441	MK099056	MK066874	MK066762
Linum	*thunbergii*	*Linopsis*	MK090442	MK099057	MK066875	MK066763
Linum	*tommasinii*	*Linum*	MK090444	MK099059	MK066877	MK066765
Linum	*tmoleum*	*Linum*	MK090443	MK099058	MK066876	MK066764
Linum	*triflorum*	*Syllinum*	MK090445	MK099060	MK066878	MK066766
Linum	*trigynum*	*Linopsis*	FJ160810	FJ160849	FJ160894	MK066767
Linum	*unguiculatum*	*Dasylinum*	MK090447	MK099061	MK066880	MK066768
Linum	*usitatissimum***	*Linum*	FJ160803	MK099062	FJ160887	MK066769
Linum	*velutinum*	*Syllinum*	MK090449	MK099063	MK066882	MK066770

Linum	*volkensii*	*Linopsis*	FJ160813	MK099067	FJ160897	MK066776
Linum	*villarianum*	*Linum*	MK090451	MK099064	MK066884	MK066772
Linum	*villosum*	*Linopsis*	MK090452	MK099065	MK066885	MK066773
Linum	*vernale**	*Linopsis*	FJ160812	FJ160851	FJ160896	MK066771
Linum	*virgatum*	*Linopsis*	MK090349	MK098984	MK066783	MK066670
Linum	*virgultorum*	*Linum*	MK090453	MK099066	MK066886	MK066774
Linum	*viscosum*	*Dasylinum*	FJ160791	FJ160830	FJ160875	MK066775

Table S2. Divergence time estimates from BEAST analysis for key nodes of the subfamily Linoideae based on partitioned data. 95% HPD = 95% highest posterior density, Ma= million years.

**Node**		**DNAcp**		**ITS**	
		Mean (Ma)	95% HPD	Mean (Ma)	95% HPD
**I**	Linoideae	41.85	36.35-48.92	44.83	36.15-56.14
**II**	*Anisadenia* + *Reinwardtia* + *Tirpitzia*	29.52	25.16-35.7	37.17	34.63-42.1
**III**	*Linum* s.l.	38.29	35.64-43	40.49	36.50-45.53
**IV**	Subclade I (sections *Dasylinum* + *Linum* + *Stellerolinum*)	31.82	25.24-38.69	33.04	24.58-42.71
**V**	Subclade II (sections *Linopsis* + *Syllinum* + *Cathartolinum* + segregated genera)	25.98	15.22-36.07	20.47	12.48-29.53
**VI**	*Hesperolinon*	3.21	1.38-5.35	4.23	2.32-6.47
**VII**	*Sclerolinon*	1.87	0.42-3.63	0.17	0-0.55
**VIII**	*Cliococca*	0.24	0-0.8	0.35	0-0.92
**IX**	*Radiola*	5.88	1.32-12.09	0.7	0.08-1.57

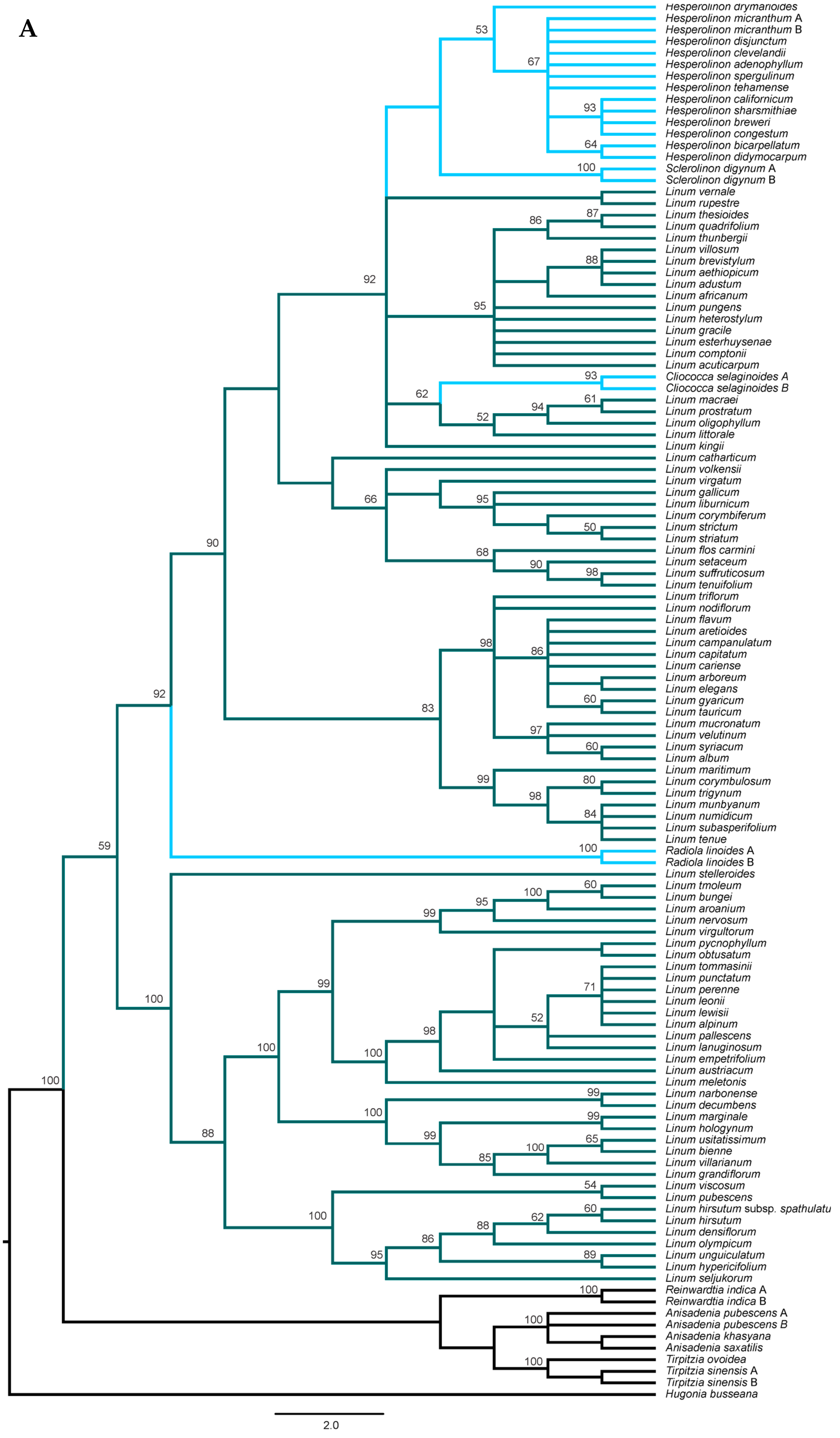
A
Hesperolinon drymarioides
Hesperolinon micranthum A
Hesperolinon micranthum B
Hesperolinon disjunctum
Hesperolinon clevelandii
Hesperolinon adenophyllum
Hesperolinon spergulinum
Hesperolinon tehamense
Hesperolinon californicum
Hesperolinon sharsmithiae
Hesperolinon breweri
Hesperolinon congestum
Hesperolinon bicarpellatum
Hesperolinon didymocarpum
Sclerolinon digynum A
Sclerolinon digynum B
Linum vernale
Linum rupestre
Linum thesioides
Linum quadrifolium
Linum thunbergii
Linum villosum
Linum brevistylum
Linum aethiopicum
Linum adustum
Linum africanum
Linum pungens
Linum heterostylum
Linum gracile
Linum esterhuysenae
Linum comptonii
Linum acuticarpum
Cliococca selaginoides A
Cliococca selaginoides B
Linum macraei
Linum prostratum
Linum oligophyllum
Linum littorale
Linum kingii
Linum catharticum
Linum volkensii
Linum virgatum
Linum gallicum
Linum liburnicum
Linum corymbiferum
Linum strictum
Linum striatum
Linum flos carmini
Linum setaceum
Linum suffruticosum
Linum tenuifolium
Linum triflorum
Linum nodiflorum
Linum flavum
Linum aretioides
Linum campanulatum
Linum capitatum
Linum cariense
Linum arboreum
Linum elegans
Linum gyaricum
Linum tauricum
Linum mucronatum
Linum velutinum
Linum syriacum
Linum album
Linum maritimum
Linum corymbulosum
Linum trigynum
Linum munbyanum
Linum numidicum
Linum subasperifolium
Linum tenue
Radiola linoides A
Radiola linoides B
Linum stelleroides
Linum tmoleum
Linum bungei
Linum aroanium
Linum nervosum
Linum virgultorum
Linum pycnophyllum
Linum obtusatum
Linum tommasinii
Linum punctatum
Linum perenne
Linum leonii
Linum lewisii
Linum alpinum
Linum pallescens
Linum lanuginosum
Linum empetrifolium
Linum austriacum
Linum meletonis
Linum narbonense
Linum decumbens
Linum marginale
Linum hologynum
Linum usitatissimum
Linum bienne
Linum villarianum
Linum grandiflorum
Linum viscosum
Linum pubescens
Linum hirsutum subsp. spathulatu
Linum hirsutum
Linum densiflorum
Linum olympicum
Linum unguiculatum
Linum hypericifolium
Linum seljukorum
Reinwardtia indica A
Reinwardtia indica B
Anisadenia pubescens A
Anisadenia pubescens B
Anisadenia khasyana
Anisadenia saxatilis
Tirpitzia ovoidea
Tirpitzia sinensis A
Tirpitzia sinensis B
Hugonia busseana
2.0

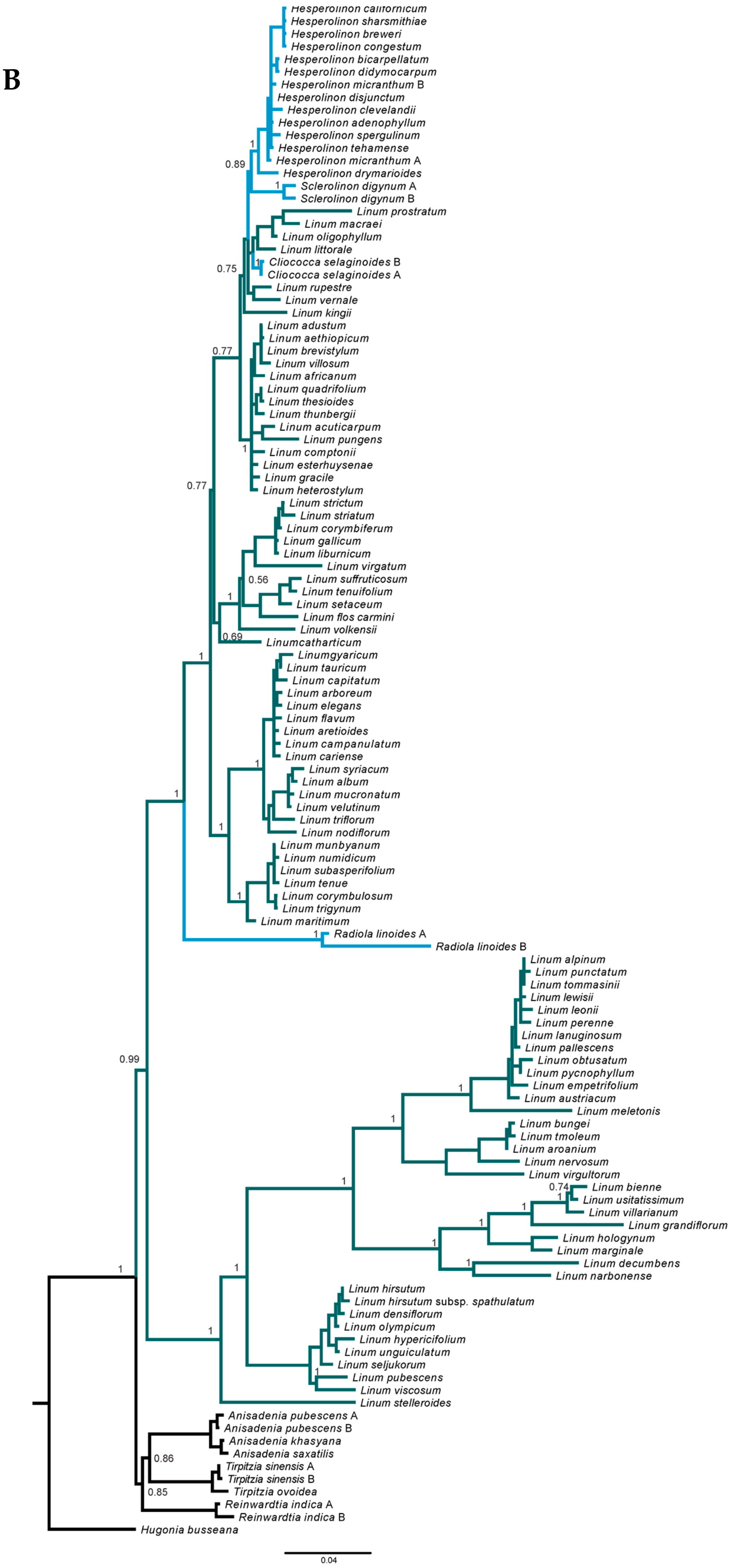

Figure S1. Phylogenetic relationships of the subfamily Linoideae based on combined plastid DNA (*ndhF*, *matK*, *trnL-F*): A) Parsimony tree. Bootstrap values (BS) are labeled above the branches. B) Bayesian Inference tree. Posterior Probabilities (PP) are labeled above the branches. *No support values. A representative of *Hugonia* (Hugonideae) was used as an outgroup.

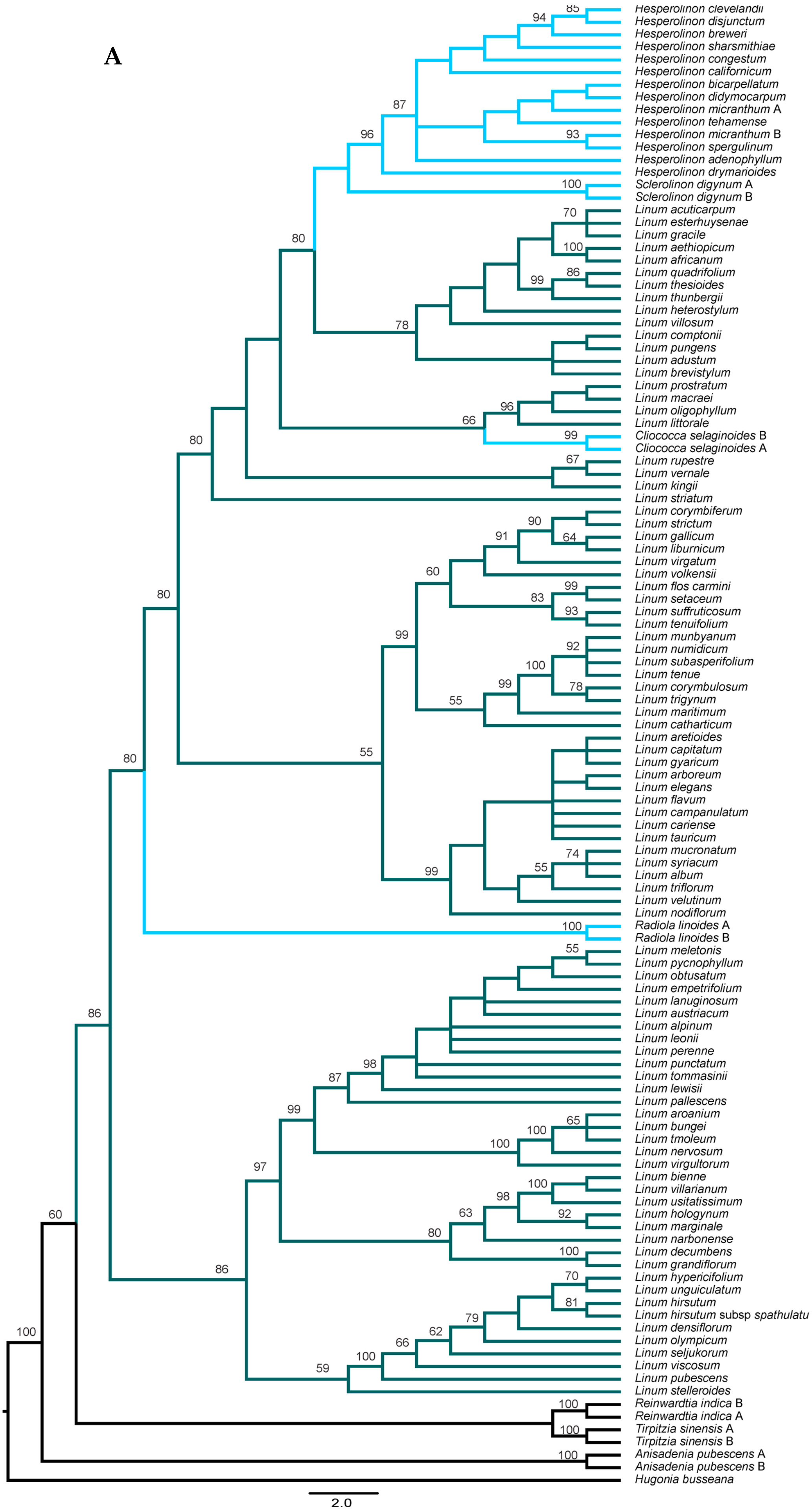
A
Hesperolinon clevelandii
Hesperolinon disjunctum
Hesperolinon breweri
Hesperolinon sharsmithiae
Hesperolinon congestum
Hesperolinon californicum
Hesperolinon bicarpellatum
Hesperolinon didymocarpum
Hesperolinon micranthum A
Hesperolinon tehamense
Hesperolinon micranthum B
Hesperolinon spergulinum
Hesperolinon adenophyllum
Hesperolinon drymarioides
Sclerolinon digynum A
Sclerolinon digynum B
Linum acuticarpum
Linum esterhuysenae
Linum gracile
Linum aethiopicum
Linum africanum
Linum quadrifolium
Linum thesioides
Linum thunbergii
Linum heterostylum
Linum villosum
Linum comptonii
Linum pungens
Linum adustum
Linum brevistylum
Linum prostratum
Linum macraei
Linum oligophyllum
Linum littorale
Cliococca selaginoides B
Cliococca selaginoides A
Linum rupestre
Linum vernale
Linum kingii
Linum striatum
Linum corymbiferum
Linum strictum
Linum gallicum
Linum liburnicum
Linum virgatum
Linum volkensii
Linum flos carmini
Linum setaceum
Linum suffruticosum
Linum tenuifolium
Linum munbyanum
Linum numidicum
Linum subasperifolium
Linum tenue
Linum corymbulosum
Linum trigynum
Linum maritimum
Linum catharticum
Linum aretioides
Linum capitatum
Linum gyaricum
Linum arboreum
Linum elegans
Linum flavum
Linum campanulatum
Linum cariense
Linum tauricum
Linum mucronatum
Linum syriacum
Linum album
Linum triflorum
Linum velutinum
Linum nodiflorum
Radiola linoides A
Radiola linoides B
Linum meletonis
Linum pycnophyllum
Linum obtusatum
Linum empetrifolium
Linum lanuginosum
Linum austriacum
Linum alpinum
Linum leonii
Linum perenne
Linum punctatum
Linum tommasinii
Linum lewisii
Linum pallescens
Linum aroanium
Linum bungei
Linum tmoleum
Linum nervosum
Linum virgultorum
Linum bienne
Linum villarianum
Linum usitatissimum
Linum hologynum
Linum marginale
Linum narbonense
Linum decumbens
Linum grandiflorum
Linum hypericifolium
Linum unguiculatum
Linum hirsutum
Linum hirsutum subsp spathulatu
Linum densiflorum
Linum olympicum
Linum seljukorum
Linum viscosum
Linum pubescens
Linum stelleroides
Reinwardtia indica B
Reinwardtia indica A
Tirpitzia sinensis A
Tirpitzia sinensis B
Anisadenia pubescens A
Anisadenia pubescens B
Hugonia busseana
2.0

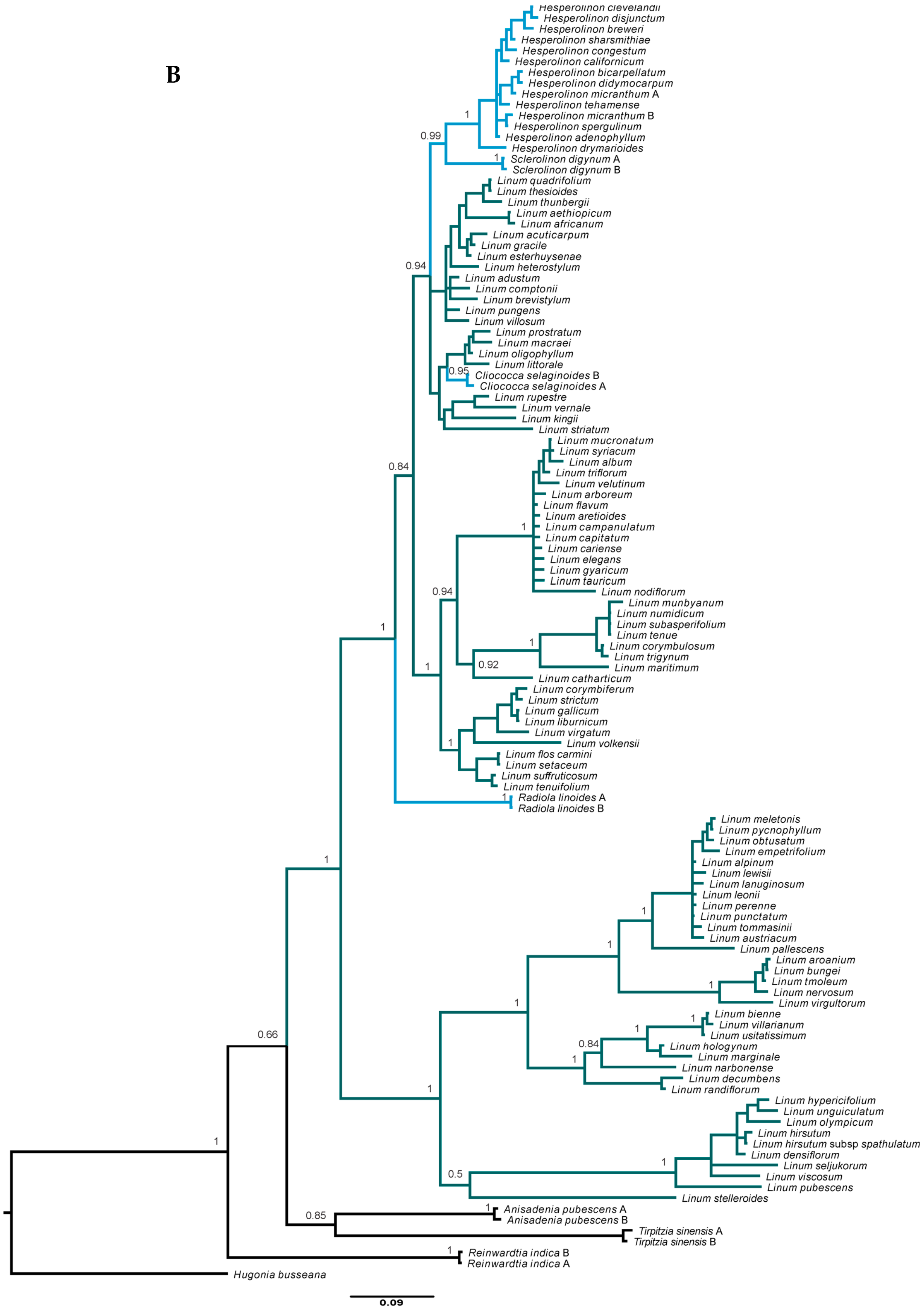

Figure S2. Phylogenetic relationships of the subfamily Linoideae based on ITS: A) Parsimony tree. Bootstrap values (BS) are labeled above the branches. B) Bayesian Inference tree. Posterior Probabilities (PP) are labeled above the branches. *No support values. A representative of *Hugonia* (Hugonideae) was used as an outgroup.

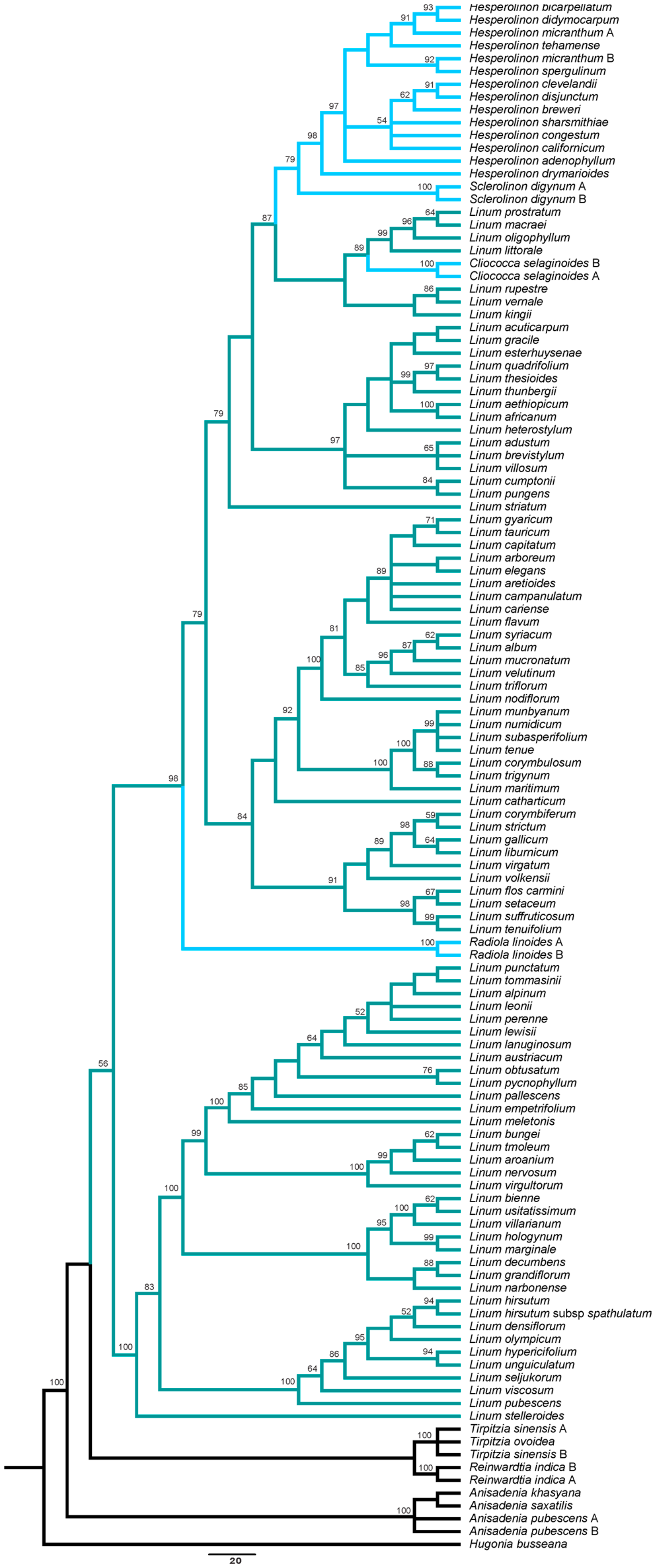

Figure S3. Parsimony Analysis tree of Linoideae based on combined plastid (*ndhF*, *matK*, *trnL-F*) and ITS dataset. Bootstrap values (BS) are labeled above the branches. *No support values. A representative of *Hugonia* (Hugonideae) was used as an outgroup.

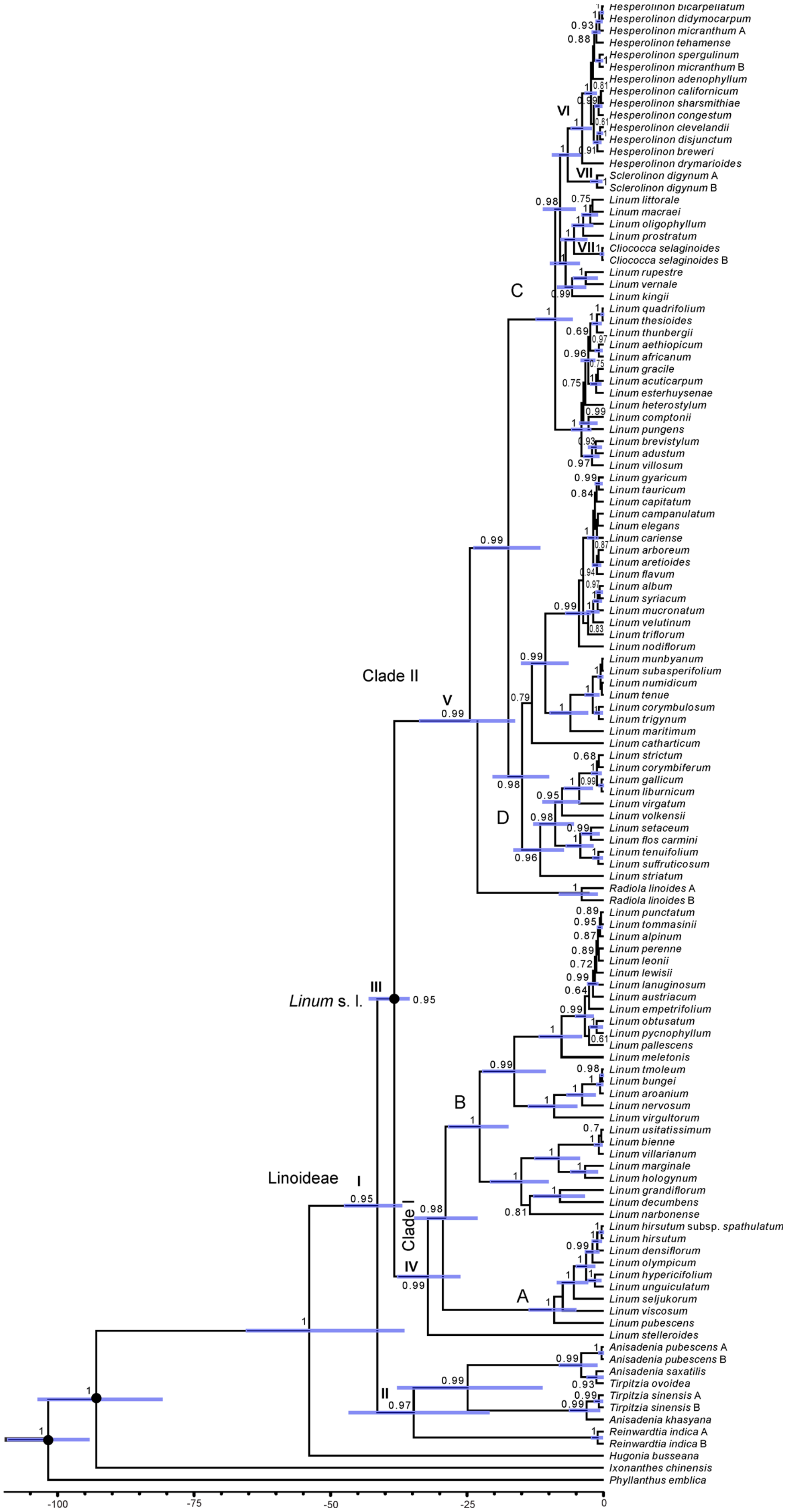

Figure S4. Beast maximum clade credibility tree of Linoideae inferred from combined ITS, *ndhF*, *matK*, *trnL-F*. Bayesian posterior probabilities > 0.6 are shown above the branches. Roman numerals below the branches indicate key nodes. Blue bars represent credibility intervals (95% highest posterior density) for the node ages. Nodes with black spots are those fixed as calibration points.

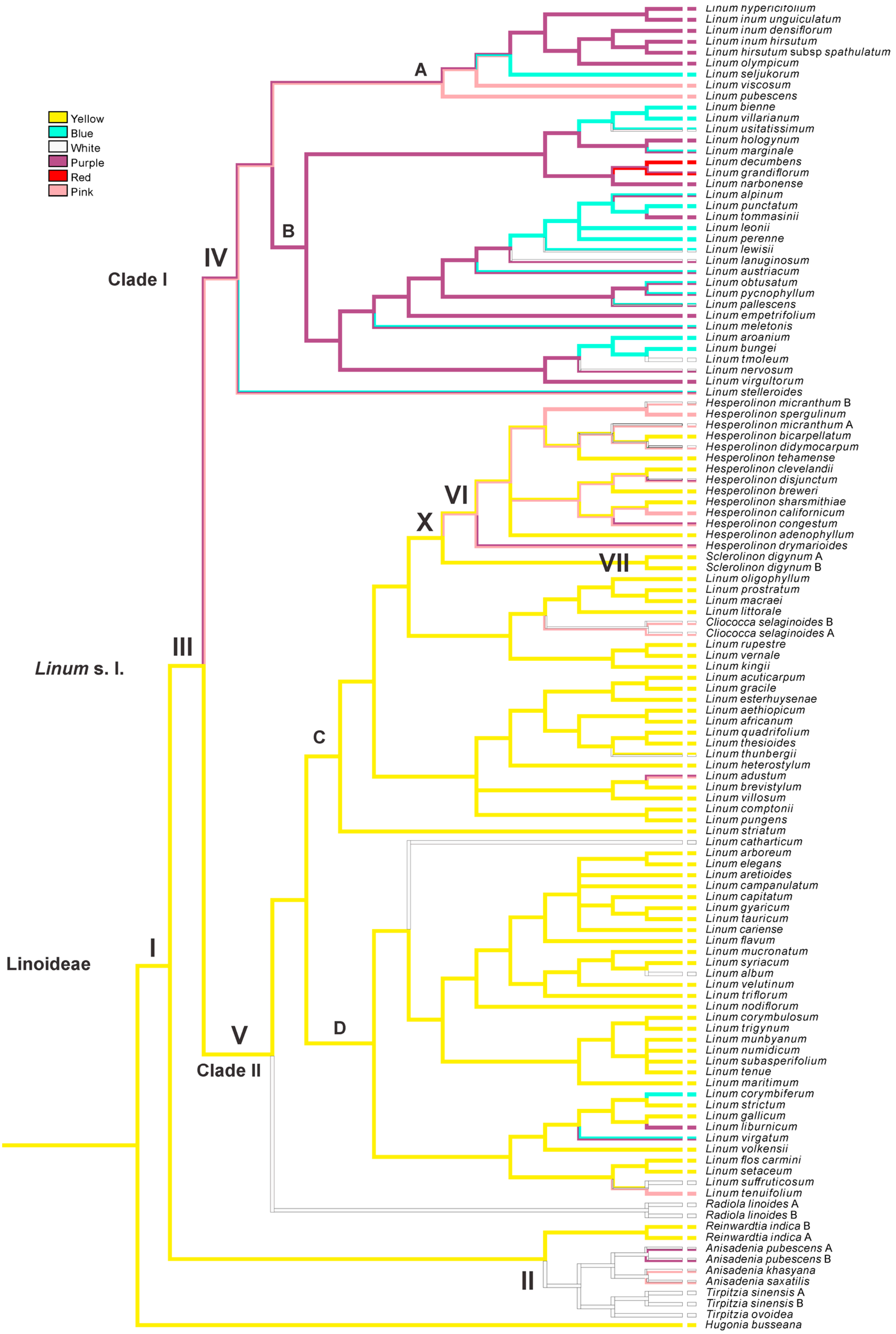

Figure S5. Reconstruction of ancestral states of flower color in the subfamily Linoideae based on Most Parsimonious Reconstruction. The colors along the branches indicate the color state estimated by Mesquite. The Roman numerals represent the key nodes. The colors at the tips represent the current color of the flower.
